# N-acetylcysteine as an adjunct to antibiotics against bacterial biofilms: A systematic review of in vitro evidence

**DOI:** 10.1016/j.bioflm.2026.100402

**Published:** 2026-09-23

**Authors:** Bakhytzhan Seksenbayev, Yerlan Suleimenov, Assylkhan Bekbayev, Nurlan Sadykov, Aziza Omari

**Affiliations:** a OLYMP Clinical Diagnostic Laboratories LLP, Astana, 010000, Kazakhstan; b School of Pharmacy, Astana Medical University, 49A Beibitshilik Street, Astana, 010000, Kazakhstan

**Keywords:** N-Acetylcysteine, Biofilm, Antibiotic combination, Formulation, pH, Antimicrobial resistance, Systematic review

## Abstract

**Background:**

N-acetylcysteine (NAC) is investigated as an antibiotic adjunct, but acidity, formulation and experimental context complicate interpretation. We evaluated comparative in-vitro evidence while distinguishing biofilm prevention, established-biofilm treatment and resistance evolution.

**Methods:**

MEDLINE/PubMed, Embase, Scopus and Web of Science were searched through August 2026, with supplementary searching. Extraction distinguished NAC preparation and exposure pH, medium, hydrodynamics, NAC-alone controls, treatment intent and interaction methodology. Results were synthesized by experimental context, regimen, phase and endpoint without pooling or whole-study efficacy voting.

**Results:**

Fifty-five studies were included: 7 evaluated combination exposure during formation only, 37 after establishment only, 9 both, and 2 resistance evolution. NAC-alone arms were documented in 39 studies, partial or formulation-specific in 9, and not identified in 7. Only two studies included a continuous-flow model. Selected combinations improved biofilm-associated outcomes, but intrinsic-acidic and neutralized preparations, biomass and viability endpoints, and planktonic versus biofilm interaction tests were not interchangeable. Liposomal and catheter-lock findings differed by dose matching, isolate and control set. Polymyxin findings varied with organism, resistance background, medium and exposure; rifampicin-alone and rifampicin-containing multidrug comparators addressed different questions.

**Conclusions:**

NAC is a context-dependent experimental adjunct, not an established class-wide antibiotic potentiator. The evidence does not support clinical antibiotic dose reduction or routine NAC combination therapy. Translation requires controlled final exposure pH, matched monotherapy and formulation controls, clinically relevant background regimens, viable-burden and regrowth outcomes, and resistance monitoring.

## 1 Introduction

Biofilms comprise attached or aggregated microorganisms embedded in an extracellular matrix. Their spatial organization and physiological heterogeneity can contribute to antimicrobial tolerance and persistence. However, poor antibiotic response is not explained by a single universal penetration barrier: the relevance of diffusion, growth state and other mechanisms depends on the organism, matrix and drug. This complexity matters across respiratory, urinary, device-associated, bone and joint, wound and dental infection settings [[1], [2], [3], [4], [5]].

N-acetylcysteine (NAC) is a thiol-containing mucolytic and antidote with several experimentally distinguishable activities. Olofsson et al. [6] demonstrated reduced extracellular polysaccharide production, altered surface wettability and reduced adhesion in a solid-surface model; those findings do not establish the same mechanism in every clinical organism [7] documented reduced extracellular polysaccharides and altered *Pseudomonas aeruginosa* biofilm architecture. Concentration-dependent direct antimicrobial effects were also observed in organism-specific experiments, including Marchese et al. [8] and Manoharan et al. [9]. Mucolytic activity against host mucus does not, however, establish cleavage of matrix-associated disulfide bonds as a general mechanism in bacterial biofilms.

Acidity is an additional experimental variable rather than an incidental property. Wang et al. [10] compared intrinsic-acidic and neutralized NAC preparations and showed that pH and bacterial resistance background influenced the response to polymyxin B. Antioxidant and redox effects provide further hypotheses, but cannot be inferred solely from a decrease in biomass or viable burden [[11], [12], [13]]. Consequently, a nominal NAC concentration does not adequately define the intervention without preparation, final exposure conditions and relevant controls.

The review question was whether adding NAC changes an antibiotic regimen's activity against bacterial biofilms relative to the corresponding regimen without NAC. The scope was not restricted to cystic fibrosis (CF), *P. aeruginosa* or medical devices. Secondary questions concerned how the findings differed by formulation, pH, medium, hydrodynamics, organism, phase, treatment intent and outcome, and whether the evidence demonstrated an incremental combination response or a formally evaluated interaction. This distinction is essential when NAC itself is active and when the antibiotic comparator differs from clinical practice.

## 2 Methods

### 2.1 Reporting framework and registration

Reporting follows PRISMA 2020 and, where applicable, Synthesis Without Meta-analysis (SWiM) guidance [14,15]. The review was not prospectively registered. Experimental-context stratification was exploratory and was not prospectively specified. The synthesis distinguished intervention chemistry, comparator attribution, biofilm phase, treatment intent and measured endpoints. Extraction definitions and interpretation rules are provided in Supplementary Section S1.

### 2.2 Eligibility and comparator hierarchy

Eligible reports were original comparative in-vitro studies of bacterial biofilms receiving NAC with one or more antibacterial antibiotics. Reference strains and clinical or veterinary isolates, mono- and multispecies systems, conventional culture models and device/material formulations were eligible. No clinical indication, organism or surface was required. Clinical-only reports, reviews, non-bacterial systems and reports without a separable biofilm-specific NAC–antibiotic comparison were excluded. Planktonic experiments were extracted only to clarify a biofilm study's interaction or mechanistic interpretation, not as independent evidence of antibiofilm efficacy.

The principal comparator was the same antibiotic or antibiotic regimen without NAC, under otherwise comparable conditions. For co-formulated or device-based interventions, interpretation required a matched antibiotic-containing carrier or background formulation without NAC. A shared additional component, such as quercetin or a companion antibiotic, was retained as part of that background, not attributed to NAC. Comparisons confounded by an unmatched active co-intervention were excluded from NAC-specific interpretation. Historically derived antibiotic-only comparisons were identified and retained as contextual observations, not treated as equivalent to concurrent controlled evidence of potentiation. Antibiotic concentration was also checked within each assay: comparisons using unequal formulation-specific MIC exposures were retained as formulation-performance context, not dose-matched evidence of NAC's incremental effect.

A NAC-alone arm was not required for eligibility because the primary question concerned the incremental effect of adding NAC to an antibiotic regimen. Its availability was systematically recorded and used to delimit inference. Without a relevant NAC-alone arm, an improvement over antibiotic alone cannot distinguish an interaction from NAC's independent activity. A NAC-alone arm does not by itself establish synergy: an appropriate interaction framework must also be applied to the relevant assay.

Formation/prevention (F) required combination exposure before or during initial biofilm development. Established-biofilm treatment (E) required exposure after a biofilm had formed; an early established biofilm was not automatically called prevention. Resistance-evolution studies (R) were analyzed separately because their main outcome was a change in susceptibility or population trajectories under selection, not acute killing. Drugs incorporated into cement or coatings before inoculation were classified as prevention regardless of the terminology in the article title. A formation experiment using NAC alone did not make an otherwise established-biofilm combination study a two-phase study.

### 2.3 Information sources and search record

Searches covered MEDLINE/PubMed, Embase via Ovid, Scopus and Web of Science Core Collection from inception through August 2026, supplemented by Google Scholar and backward/forward citation searching. Strategies combined acetylcysteine/NAC, antibacterial agents and biofilm/sessile-cell concepts. Database strategies and recorded yields are presented in Supplementary Section S2. Exact database-specific execution dates, raw export files and saved search histories were unavailable for verification. In particular, the reported PubMed yield of two records could not be independently reconciled with a search history. This gap limits reproducibility and does not establish that PubMed contained only two relevant records.

### 2.4 Selection and source verification

After deduplication, titles and abstracts were screened against the predefined eligibility criteria. Potentially eligible reports were then assessed in full text against the eligibility criteria and comparator hierarchy. Screening and primary data extraction were not performed independently in duplicate.

Full texts of all 55 included reports were examined during source verification, including the Methods sections, numerical tables, figures, and relevant figure annotations. Extracted information and eligibility decisions were checked against the source publications, with particular attention to study design, treatment phase, comparators, NAC formulation and concentration, exposure conditions, and reported outcomes. Inconsistencies or ambiguities within source reports were recorded explicitly rather than resolved by assumption.

Full-text exclusions and reports that could not be retrieved are documented in Supplementary Section S3. Retrieval status was distinguished from the availability of source documents used to verify extracted information; the absence of a source copy was not, by itself, treated as evidence for an eligibility decision.

### 2.5 Extraction and classification

Data extraction recorded bibliographic details, organism and isolate information, substrate, biofilm age and phase, antibiotic regimen, NAC concentration and formulation, comparator, measured outcomes and treatment intent. Experimental-context fields separately recorded stock preparation and pH, final exposure pH, pH controls, growth medium, treatment medium and hydrodynamics. Static batch, agitated batch and continuous flow were distinguished; periodic medium replacement was not classified as flow. Values belonging only to washing, staining, chromatography, release or dissolution experiments were not used as treatment-pH measurements.

NAC-alone coverage was coded as documented, partial/formulation-specific, not identified in the relevant biofilm contrast, or not assessable. “Not identified” is not proof that an arm never existed. Formulation-specific controls were not equated with a complete free-drug factorial experiment. Treatment intent was recorded separately as prevention/anti-adhesion, viable-cell reduction, matrix/biomass disruption, delivery enhancement or resistance modulation; studies could address more than one intention. Clinical context was assigned from the source's stated setting or design rather than inferred from the organism.

Study-level context and phase records are supplied for all included studies. A focused table of 25 comparisons separates particularly important pH, medium, regimen, dose-matching and endpoint contrasts, including distinct assays within a report. This is not an exhaustive strain-by-concentration arm-level database. Selected explicitly reported numerical values are transcribed with their original units and caveats in a separate data file. Missing numerical effects or variances were not imputed, graphical values were not digitized, and inconsistent SD/SEM labels were not converted into effect-size variances. Dose ranges and assay details remain linked to the source rather than being converted into a purported common exposure scale.

### 2.6 Appraisal of interpretability

Interpretability was appraised by domain: comparator attribution, NAC-alone coverage, exposure definition, medium and hydrodynamics, phase–endpoint alignment, interaction methodology and source-verification completeness. A report describing stock pH was not necessarily considered pH-controlled during treatment. Replication and uncertainty statements were checked where relevant to the focused comparisons. The appraisal did not use a summed reporting score or a validated risk-of-bias instrument. Internal validity and clinical applicability were considered separately: a controlled formulation experiment may be internally interpretable yet remote from systemic treatment.

### 2.7 Synthesis and robustness assessment

A structured narrative synthesis groups observations by phase, organism/regimen, pH/formulation, medium and hydrodynamics, then distinguishes viability, biomass, susceptibility-threshold, structural and evolutionary endpoints. A single favorable or unfavorable label was not assigned to an entire paper, because different drugs, phases and concentrations within that paper can disagree. Statistical significance was not used to count “positive studies,” and failure to reach significance was not interpreted as proof of equivalence. Reported synergy was distinguished from the review's description of an incremental response, and a planktonic fractional inhibitory concentration index (FICI) was not transferred to a biofilm endpoint.

Meta-analysis was not performed because outcome definitions and exposure conditions were not sufficiently commensurate and the extraction did not provide a complete independent effect-size/variance dataset. Descriptive counts characterize evidence coverage, not treatment success rates. Each study contributes once to a mutually exclusive phase group; studies with both F and E contribute to each phase-component total, without being treated as independent experiments. Robustness was examined qualitatively by excluding unequal-dose, historical-comparator or inadequately controlled comparisons from strong mechanistic claims. Reproducible CSV datasets and Python code generate the descriptive summaries and figures. No pooled efficacy estimate, formal subgroup interaction test or publication-bias test was calculated.

## 3 Results

### 3.1 Study selection and source coverage

The selection record contained 1021 identified records. After removal of 514 duplicates, 507 records underwent title and abstract screening, of which 437 were excluded. Seventy reports were sought for full-text assessment; five were not retrieved. Of the 65 reports assessed, 10 were excluded and 55 were included (Fig. 1). Identification and screening counts could not be verified against raw search exports, as described in Section 2.3.

**Fig. 1:**
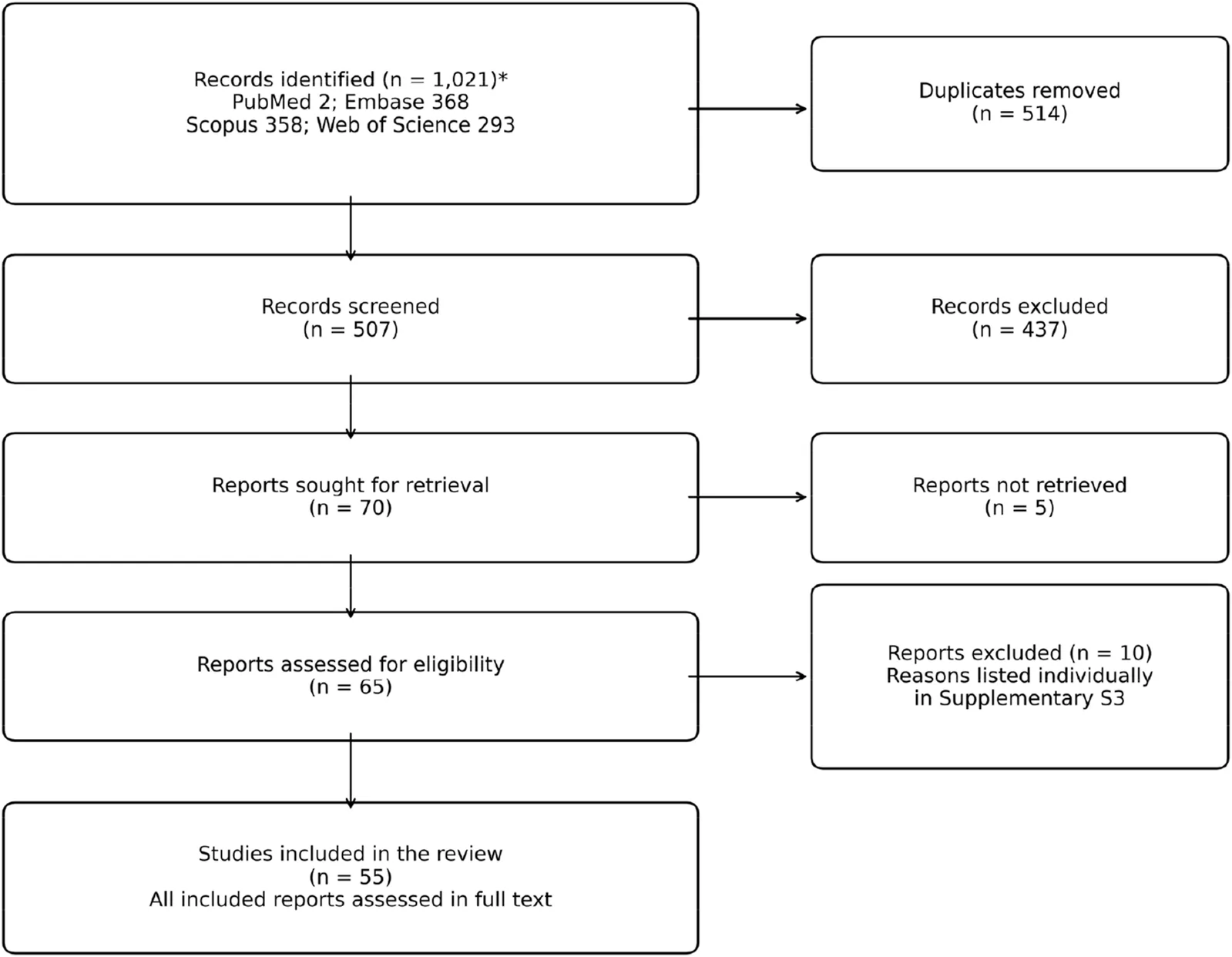
Study identification and selection. Of 1021 identified records, 514 duplicates were removed and 507 records were screened. Seventy reports were sought, five were not retrieved, and 65 were assessed in full text. Ten reports were excluded and 55 studies were included. All included reports were assessed in full text. Identification and screening counts could not be verified against raw search exports; exact search dates and the separate contribution of supplementary searching were unavailable.

The 55 included reports spanned 2003–2026, and full texts were available for all included studies. Evidence coverage is summarized in Table 1; study-level characteristics, source locators and interpretive notes are provided in Supplementary Section S4.

### 3.2 Biofilm phase, intent and clinical context

Seven studies evaluated combination exposure during formation only, 37 after establishment only and nine both; two focused on resistance evolution. Thus, 16 studies contributed acute formation components and 46 established-biofilm components; the two evolutionary studies were summarized separately (Fig. 2). These classifications follow actual exposure timing rather than the words “inhibition,” “development” or “eradication” in titles or assay labels. For example, pretreatment of cement belongs to prevention, whereas a post-formation minimum biofilm inhibitory concentration assay remains an established-biofilm experiment.

**Fig. 2:**
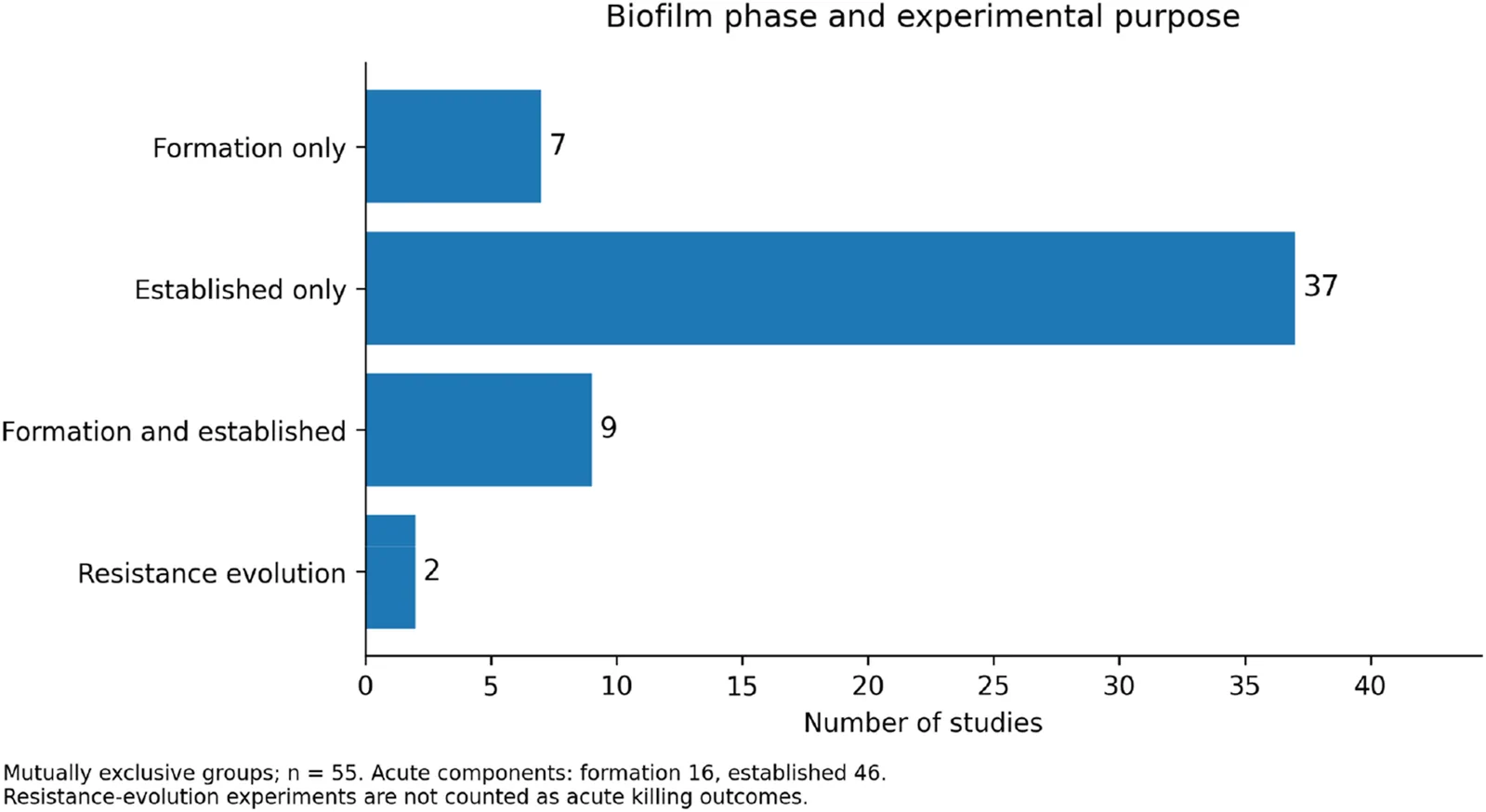
Biofilm phase and experimental purpose. Mutually exclusive groups: formation only, established only, both phases, and resistance evolution. Formation and established components total 16 and 46, respectively. Counts describe coverage, not efficacy.

The clinical-context map includes urinary/catheter and uropathogenic-isolate studies [8,9,[16], [17], [18], [19]], orthopedic/cement and shunt models [[20], [21], [22], [23], [24]], dental/periodontal systems [25,26], veterinary isolates ([27]; Son et al., 2021; [28]), and respiratory/CF-motivated models [[29], [30], [31], [32], [33]]. CF and local/device delivery are prominent research contexts, but they are neither the eligibility boundary nor a proxy for all biofilm infections. The source-linked context field is reported for every study; these experimental settings should not be read as evidence of clinical effectiveness in those patient populations.

### 3.3 NAC controls and experimental reporting

A NAC-alone arm was documented in 39 studies; nine had partial or formulation-specific coverage and seven had no such arm identified in the relevant contrast. All 55 reports were assessable for control documentation (Fig. 3). These are documentation counts, not a claim that every dose and endpoint had a complete factorial control set. Alarfaj et al. [34] included free NAC but did not report a NAC-only liposome. Mansouri et al. [19] included both single agents in its 12-h assay, but not in the extended durability assay. In Aksoy et al. [18], the additional response cannot be partitioned into direct NAC activity and interaction with each antibiotic.

**Fig. 3:**
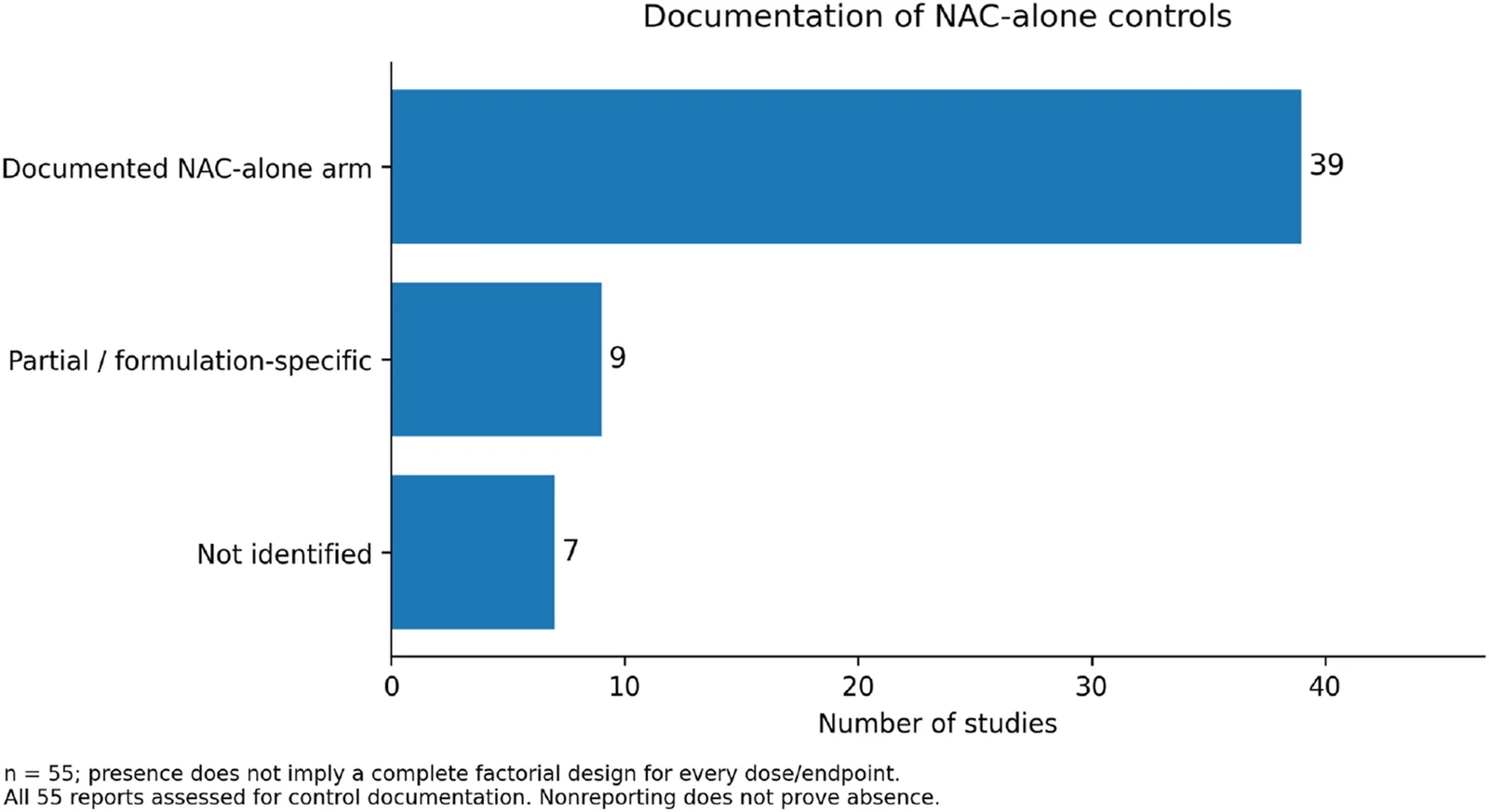
Documentation of NAC-alone controls. A NAC-alone arm was documented in 39 studies, partial or formulation-specific in nine, and not identified in the relevant contrast in seven. All 55 reports were assessable for control documentation. These counts do not establish a complete factorial design for every endpoint or formal synergy.

Eleven studies explicitly used static batch models, 15 agitated or mixed batch conditions, 27 batch conditions without clear agitation reporting, and two included continuous flow (Fig. 4). Mansouri et al. [19] combined a stationary catheter-lock dwell with rocker-grown SEM specimens and was therefore classified as mixed batch, not flow. Birk et al. [31] studied a coating/delivery strategy under artificial-sputum flow, whereas Higazy et al. [12] included flow-cell and bead-based resistance models. Their different interventions and endpoints prevent a flow-versus-static efficacy conclusion. Unspecified agitation was not classified as static.

**Fig. 4:**
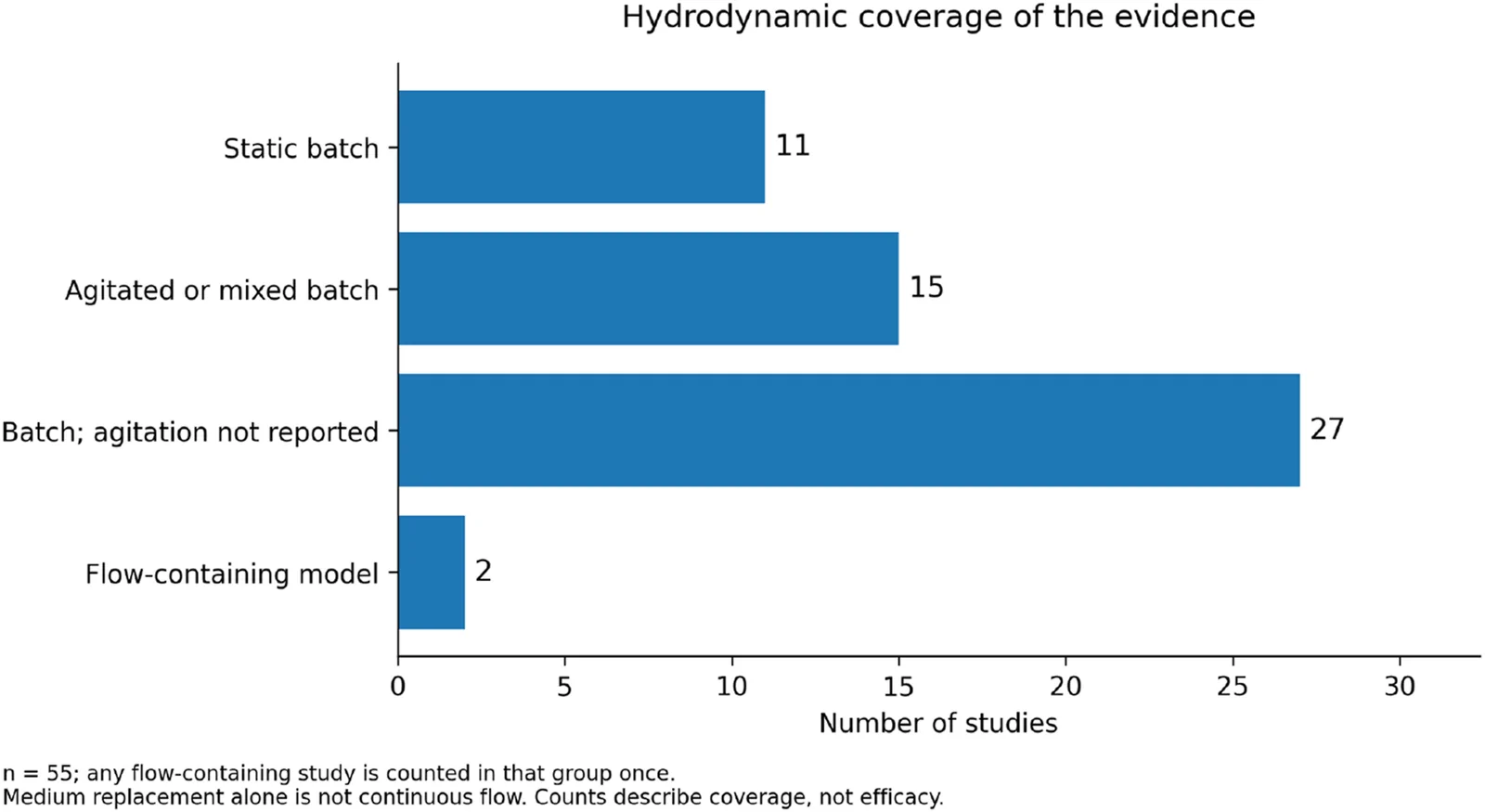
Hydrodynamic coverage. Any study with a continuous-flow component is counted once in the flow-containing group. Agitated/mixed batch is separate from explicitly static and unspecified batch. No efficacy comparison of model types is inferred.

Preparation pH, final exposure pH and pH-matched controls were distinct features of the evidence [7] reported stock adjustment to pH 7.2; Pollini et al. [35] and Ciacci et al. [36] reported adjustment to 6.5–6.8. These preparation details do not establish final per-well pH control. Alarfaj et al. [34] used phosphate-buffered saline (PBS) nominally at pH 7.4 for preparation, without a separately reported final NAC-containing biofilm-exposure pH. Onger et al. [20] and Mansouri et al. [19] did not report exposure pH or an acid-only/pH-matched control. PBS used only for washing, and pH values from formulation release or analytical procedures, did not establish treatment pH. Preparation, exposure and control information were therefore reported separately rather than collapsed into a binary pH-control indicator.

### 3.4 pH-, medium- and formulation-stratified findings

Intrinsic-acidic NAC exposures require separate interpretation from neutralized stocks (Table 2). Aiyer et al. [29] examined acidic NAC and antibiotic treatment of *Burkholderia cenocepacia* biofilms, including measured pH under PBS/TSB conditions. NAC-alone activity, acid exposure and the treatment vehicle are part of that intervention. Aiyer et al. [30], by contrast, adjusted NAC stock to a near-neutral range before testing combinations against three emerging CF pathogens. Differences between those reports cannot be attributed solely to NAC concentration or used as a controlled comparison of pH, because organisms and other assay conditions also differ.

Medium-specific comparisons within Valzano et al. [32] are especially informative. Neutralized-stock NAC–colistin experiments used both conventional broth/peg and artificial-sputum conditions. In selected artificial-sputum experiments, the strong combination response occurred at colistin 64 mg/L with NAC 8 g/L; lower tested colistin exposures did not reproduce that response. This is not evidence that NAC permits clinically reduced polymyxin dosing. It demonstrates why exposure medium, organism background and concentration must remain attached to each observation rather than combined into a class-wide estimate.

Wang et al. [10] compared intrinsic-acidic and neutralized NAC conditions in *Klebsiella pneumoniae* and found resistance-background-dependent effects. Its planktonic interaction analysis and crystal-violet formation assay answer different questions. The reported additive interactions in selected nonsusceptible groups do not establish synergy against established biofilms. Similarly, an altered matrix signal in a free-drug assay is not interchangeable with a change in release, retention or surface behavior in a nanoparticle, powder or coating formulation.

### 3.5 Prevention, anti-adhesion and biomass endpoints

Prevention evidence included cement or surface pretreatment, growth in the presence of agents, and early formation assays [37,38]. These designs test reduced attachment or accumulation under exposure, not removal of an existing infection. The NAC/cement experiments of Onger et al. [20] and Gocer et al. [21] were therefore classified as prevention. Espinosa et al. [27] separately tested formation and biofilms aged 4, 8 and 24 h; effects depended on biofilm age and did not demonstrate sustained elimination of older biofilms.

In Onger et al. [20], cement contained 6 mg NAC and/or 2 g ciprofloxacin per 40 g before 48-h colonization in TSB with 0.5% glucose. The four groups each contained six molds. The reported combination count was numerically lower than the ciprofloxacin-only count, but the article's preformed-biofilm/eradication language is not accompanied by a separate delayed-treatment protocol. Table 1 also mixes SD/SEM labels, and the Results do not explicitly report the antibiotic-only versus combination pairwise statistic. Accordingly, this is formulation-specific prevention/colonization evidence, not proof of complete eradication or a quantified free-drug interaction. The loading amount is not a measured eluted NAC concentration (Supplementary S4–S5, C21).

**Table 1:** Evidence coverage and experimental classification.

Domain	Result	Interpretive note
Included studies	55	Full texts available and assessed for all 55 included reports
Phase groups	F only 7; E only 37; F + E 9; R 2	F: combination during formation; E: after establishment; R: resistance evolution
Acute phase components	Formation 16; established 46	The 9 two-phase studies contribute to both totals; R is separate
NAC-alone control coverage	39 documented; 9 partial; 7 not identified; 0 not assessable	Documented does not mean every dose/endpoint had full factorial controls
Hydrodynamics	11 static; 15 agitated/mixed; 27 batch unspecified; 2 flow-containing	Recovery vortexing and medium replacement are not flow; separate rocker and lock assays count as mixed batch
Exposure pH	Separate stock/exposure/control fields	Preparation, exposure and pH-matched control information are distinguished
Clinical context	Respiratory, urinary, device/implant, dental, veterinary and other models	Context is recorded per study; no indication restriction was imposed

**Table 2:** Experimental-context strata and implications.

Stratum	Examples	Interpretation
Intrinsic-acidic exposure	[29] [10];	NAC alone and acidity may contribute to the response; these exposures are not interchangeable with neutralized preparations.
Adjusted stock; final exposure incompletely defined	[7] (7.2) [35];/[36] (6.5–6.8)	The stock pH is documented; final per-well pH and pH-matched controls are separate questions.
Medium changes within a study	[32]: CAMHB peg versus artificial sputum	Selected strong sputum responses required high colistin exposure. These are not interchangeable dose-sparing observations.
Flow-dependent delivery	[31]: artificial sputum; coating; continuous flow	Matched coating/formulation comparison; not free-drug synergy or a controlled flow-versus-static trial.
Formation/planktonic interaction split	[10,39]	Formation responses cannot be called mature-biofilm killing. Planktonic FICI does not establish biofilm synergy.
Missing NAC-alone biofilm contrast	[18]; NAC 75 mg/mL; CAMHB	Residual biomass/author-defined MBIC change cannot partition direct NAC action from interaction.
Non-exposure pH values	Washing, staining, chromatography, release and dissolution procedures	Not assigned as final biofilm treatment pH. Study-specific examples are in the extraction notes.
Dose-matched versus unequal formulation exposure	[34]; TL versus TNL	Only EC_219 and KP_059 use equal reported tobramycin doses; their biomass directions differ. Four other biofilm contrasts use unequal doses.
Assay-specific control sets	[19]; 12-h lock versus 24–96-h durability	Both single agents are present in the main assay, not the durability assay. VRE/Klebsiella added-benefit contrasts are nonsignificant.
Preloaded cement; inconsistent eradication framing	[20]; 6 mg NAC and 2 g ciprofloxacin per 40 g cement	Colonization under drug exposure is prevention. Timing and SD/SEM ambiguities limit interpretation; loading is not a measured dissolved dose.

Lima et al. [40] applied a combination-response framework to a formation/biomass endpoint with tobramycin. Such an interaction result remains specific to that endpoint. Petkova et al. [28] examined doxycycline-associated formation responses under agitated then static batch conditions; the findings do not support a universal antibiotic-potentiating action. Son et al. [39] did not establish significant synergy in its canine *P. aeruginosa* combination experiments, including the polymyxin B comparison. These prevention findings do not establish efficacy against mature biofilms.

Biomass stains measure retained material rather than bacterial killing. Lower crystal-violet or safranin signals may reflect reduced growth, attachment, matrix abundance or detachment. Without viable counts and recovery or regrowth assessment, an anti-adhesion or dispersal response does not establish bactericidal eradication. In Aksoy et al. [18], catheter-derived Gram-negative isolates formed 24-h microplate biofilms before exposure to antibiotics with or without NAC 75 mg/mL. The crystal-violet endpoint represents residual biomass, and a NAC-alone biofilm arm was not identified. The clinical origin of the isolates does not make the microplate assay a catheter-surface experiment. Endpoint-specific interpretation also applies to the formation and detachment components of formulation studies such as Josef et al. [41].

### 3.6 Established-biofilm treatment and antibiotic backgrounds

Established-biofilm evidence encompassed viable counts, metabolic estimates, MBEC/MBIC-type thresholds and structural outcomes. Selected positive responses were reported across antibiotic classes, but they were dependent on drug, strain, concentration and assay [42 [42], [43], [44], [45], [46], [47]]. Examples include fosfomycin against uropathogenic *Escherichia coli* [8], ciprofloxacin with *P. aeruginosa* [7], linezolid or vancomycin with *Staphylococcus epidermidis* [30,48,49], and cefditoren with *Streptococcus pneumoniae* [50]. A mechanistic interpretation cannot be transferred between these organisms or endpoint types.

Mansouri et al. [19] treated 24-h catheter biofilms for 12 h with NAC 10 mg/mL, levofloxacin 5 mg/mL, their combination or saline. Fig. 1 of that report supports lower combination CFU than levofloxacin alone for MRSE, levofloxacin-sensitive and -resistant MRSA, *P. aeruginosa* and *E. coli*. However, the levofloxacin-versus-combination contrasts for VRE and *K. pneumoniae* are explicitly marked nonsignificant; this does not establish equivalence. The source tested two MRSA susceptibility phenotypes, not an MSSA group. These are matched-concentration incremental comparisons without a formal interaction model (C24).

The same report's 24–96-h durability experiment compared the combination only with saline, not either monotherapy. No *P. aeruginosa* or *E. coli* colonies were recovered at 24 h and no MRSA colonies at 96 h, whereas MRSE remained recoverable. These results cannot establish antibiotic-relative durability or clinical catheter salvage. The nutrient-poor saline environment, unreported exposure pH, absence of a stated quantitative detection limit and lack of post-withdrawal regrowth testing constrain the interpretation. Catheter force-at-break testing assessed material compatibility, not patient safety ([19]; C25).

The rifampicin evidence was divided by background regimen (Table 3). Leite et al. [51] compared rifampicin with and without NAC in an agitated *S. epidermidis* model. Belfield et al. [52] included a rifampicin–clindamycin background for staphylococci, and the relevant Pijls et al. [24] comparison used vancomycin–rifampicin with or without NAC under matched no-heat conditions. Benefits from induction heating in other Pijls arms were not attributed to NAC. Rifampicin/clindamycin-impregnated shunts in Kuruoglu et al. [23] were likewise treated as a background device factor rather than a rifampicin monotherapy experiment.

**Table 3:** Comparator backgrounds relevant to rifampicin and attribution.

Background comparison	Examples	What the contrast establishes
Rifampicin ± NAC	[51]	Mechanistic monotherapy comparator; limited direct clinical-regimen relevance
Rifampicin + clindamycin ± NAC	[52]	Incremental NAC response on a two-antibiotic background
Vancomycin + rifampicin ± NAC, without heating	[24]	Matched no-heat contrast; heating effects in other arms are not attributed to NAC
Antibiotic-impregnated shunt background	[23]	Matched shunt type and background antibiotics are required; this is not a free rifampicin-alone comparison.
Historical antibiotic-only comparisons	[53,54]	Contextual evidence, not interchangeable with concurrent factorial control

Molina-Manso et al. [53] and Muñoz-Egea et al. [54] incorporated previously published antibiotic-only results for some comparisons. Such observations can reveal hypotheses or inconsistency, but do not provide the same protection against batch and protocol differences as concurrent controls. They were retained with explicit provenance and were not used as independent confirmation of potentiation. Muñoz-Egea's exposure after biofilm development was classified as established treatment, even though the article also examined developmental architecture.

### 3.7 Organism and resistance-background heterogeneity

The polymyxin-related evidence spans *P. aeruginosa*, *Acinetobacter baumannii*, *Stenotrophomonas maltophilia* and *K. pneumoniae*, with different susceptibility backgrounds and assay purposes [10,30,32,35,36,39]. Aksoy et al. [18] also evaluated colistin among six antibiotics. Selected established-biofilm responses coexist with neutral or conditional findings and with prevention-only evidence. Differences in organism, resistance background, medium, exposure and endpoint preclude a class-wide conclusion about the consistency of potentiation.

Resistance-related results were analyzed independently of acute antibiofilm treatment. Higazy et al. [12,13] investigated evolutionary responses under antibiotic exposure in different biofilm/aggregate environments. NAC's influence was model- and exposure-dependent; these experiments do not establish that NAC universally prevents resistance. Nor can an evolutionary outcome be combined numerically with a reduction in stained biomass or an immediate viable-count change.

### 3.8 Formulation and delivery effects

Liposomal, nanoparticle, cyclodextrin, powder, coating and device systems alter more than the chemical presence of NAC [23,31,33,34,41,[55], [56], [57], [58], [59], [60]]. The extraction therefore identifies the actual matched formulation and any shared active background. For example, the ciprofloxacin–quercetin versus ciprofloxacin–quercetin–NAC contrast in Alhajj et al. [33] concerns a ternary delivery system, not an isolated free NAC–ciprofloxacin interaction. These reports support formulation-specific hypotheses rather than a formulation-independent NAC effect.

Alarfaj et al. [34] illustrates the importance of dose matching. Its six biofilm figures tested liposomal tobramycin (TL) and tobramycin–NAC liposomes (TNL) at their respective MICs; only EC_219 (32 mg/L in both arms) and KP_059 (16 mg/L in both) had equal reported tobramycin doses. TNL's mean residual-biomass bar was higher than TL's for EC_219 but lower for KP_059, without a displayed TL-versus-TNL pairwise significance test. The other four isolate comparisons used unequal doses and cannot isolate an incremental NAC effect. Free NAC 50 mM was included, but a NAC-only liposome was not reported, and particle size/encapsulation differed. Nominal preparation NAC, planktonic MIC improvement and lower crystal-violet biomass therefore do not establish a uniform NAC effect or biofilm synergy. The inconsistent 72-h/additional-48-h growth chronology is preserved in the extraction (C22–C23).

### 3.9 Drug-specific absence of benefit and adverse interactions

Drug-specific unfavorable and neutral observations were retained [61,62]. Moon et al. [25] described reduced activity with ampicillin, ciprofloxacin and tetracycline under the tested conditions, whereas this antagonistic pattern was not reported for metronidazole. Neutral or strain-dependent findings in staphylococcal and mycobacterial models likewise caution against assuming that matrix modification necessarily improves antibiotic activity [53,54,63].

### 3.10 Robustness of the interpretation

The experimental qualifications were consequential to interpretation. Onger's loading chronology and variance labels, Alarfaj's unequal formulation doses and opposite dose-matched biomass directions, and Mansouri's organism- and assay-specific controls supported different levels of attribution. Historical-comparator and unequal-dose observations were excluded from strong claims of drug interaction. Restricting interpretation to appropriately matched comparisons did not support a universal NAC effect or a cross-class ranking. This assessment was qualitative; no pooled sensitivity estimate was calculated.

## 4 Discussion

### 4.1 Principal interpretation

The principal finding is context-dependent experimental activity rather than a general antibiotic-potentiating property. Adding NAC sometimes improves a biofilm-associated outcome [64], but that response may reflect direct antimicrobial action, altered adhesion or matrix, acidity, changes in delivery, or a drug interaction. These mechanisms cannot be distinguished solely by the direction of an outcome. The evidence from respiratory, non-CF, veterinary, dental and device-based models therefore supports interpretation at the level of the specific intervention, comparator and endpoint.

### 4.2 Formulation, pH and medium are part of the intervention

Nominally equal concentrations of NAC can represent different exposures when stock neutralization, treatment medium and buffering differ. Conversely, different concentrations cannot be treated as a common dose–response series when one intervention is intrinsically acidic and another is neutralized. A neutralized stock also does not guarantee a stable neutral environment after mixing, bacterial growth or incubation. Future factorial experiments should measure final pH before and after exposure and compare untreated, acid-only, NAC-alone, antibiotic-alone and combination conditions with matched vehicles and ionic strength. Buffering should not be assumed from a medium name.

NAC-associated reductions in extracellular polysaccharides or attachment are experimentally supported in particular systems, but the operative mechanism must be tested rather than inherited from NAC's mucolytic reputation. Olofsson et al. [6], for example, distinguished reduced polysaccharide production from degradation of existing polysaccharide. Acidity-sensitive responses in Wang et al. [10] and neutralized-stock biofilm studies support retaining both physicochemical and organism-specific explanations; neither excludes other mechanisms universally.

### 4.3 Biological meaning and clinical context

Prevention, dispersal and killing require different evidence. A coating that reduces initial colonization may be useful without killing an established community, whereas loss of attached biomass can release viable organisms. Translation therefore requires testing the detached fraction and regrowth as well as retained biomass. Similarly, an MBEC or MBIC label is not sufficient to establish a standardized outcome; inoculation timing, recovery conditions, threshold and detection limit must be described. Biofilm susceptibility thresholds should not automatically be interpreted with planktonic clinical breakpoints.

Respiratory mucus and local/device delivery are prominent in the mapped evidence, but that concentration is a limitation of the eligible comparative evidence, not proof that the wider NAC literature is confined to these contexts. The review excluded many NAC-only and unmatched formulation experiments because they could not answer the incremental-antibiotic question. Non-CF urinary, dental, orthopedic and veterinary examples broaden the map but do not establish generalizability to wounds, systemic infections or other tissues. Studies selected for those specific environments remain necessary.

### 4.4 Polymyxins: exposure, toxicity and stewardship

Polymyxins are reserve or alternative agents in selected resistant Gram-negative infections, not a uniformly preferred background therapy. Current IDSA guidance describes important limitations in systemic exposure and an extremely narrow therapeutic window associated with nephrotoxicity, including in carbapenem-resistant *A. baumannii* treatment [65]. A favorable in-vitro NAC contrast must therefore not be translated into empiric polymyxin dose reduction. The sputum-model concentration dependence in Valzano et al. [32] particularly argues against such an inference.

Sublethal exposure, regrowth and survival of resistant or heteroresistant subpopulations are stewardship concerns to test prospectively, not demonstrated benefits or harms of NAC from this review. An early reduction in bulk burden would be insufficient to resolve them. Studies should retain clinically appropriate background exposure, characterize susceptible and resistant subpopulations, assess regrowth after withdrawal, and measure changes in susceptibility during serial treatment. Separate organism- and strain-level analyses are needed: a response in an *A. baumannii* peg model cannot predict the response of *K. pneumoniae* under acidic formation conditions. The available NAC evidence does not establish a safe polymyxin-sparing strategy.

### 4.5 Rifampicin and clinically relevant combination comparators

For active staphylococcal implant-associated infection, rifampicin is used with companion antibiotics because monotherapy readily selects resistance. The IDSA prosthetic-joint infection guideline explicitly places rifampicin within combination regimens in the relevant management settings [66]. Thus, rifampicin-alone experiments can characterize an incremental in-vitro response but are not direct tests against a clinically appropriate treatment regimen. A small NAC increment when rifampicin alone is already active should not be generalized to absence of benefit on a multidrug background.

The clinically informative question is whether NAC adds sustained benefit to an otherwise appropriate complete regimen, without compromising companion-drug activity or resistance suppression. Belfield et al. [52] and the no-heat multidrug comparison in Pijls et al. [24] are closer to that comparator structure, although still in vitro. NAC is not established as a substitute for an active companion antibiotic. The same principle applies to other settings in which routine treatment is multidrug: antibiotic-plus-NAC versus a single antibiotic cannot, by itself, predict the effect of adding NAC to standard care.

### 4.6 Strengths and limitations

Strengths include retention of negative and neutral findings, source-linked extraction of experimental context, separation of stock and exposure pH, explicit assessment of monotherapy-control coverage, and interpretation at the study-phase and comparison levels. All included reports were available in full text, allowing the synthesis to distinguish methodological descriptions from broader therapeutic claims.

Several limitations constrain the review. Raw search exports, exact execution dates and the very low reported PubMed yield could not be verified, so the completeness and reproducibility of the search cannot be assured. Despite full-text availability for all included studies, source reporting left unresolved pH, dose, timing and uncertainty details, and some antibiotic-only comparisons were historically derived rather than concurrent. Experimental-context stratification was exploratory rather than prospectively specified. “Not reported” denotes missing source information, not demonstrated absence of a control. Full-text availability does not ensure complete or internally consistent reporting.

The extraction provides study/phase summaries and focused comparison records, not a complete arm-level dataset. Consequently, effect magnitudes were not pooled, concentration–response surfaces were not reconstructed, and no statistical subgroup or publication-bias analysis was possible. Descriptive coverage counts do not measure evidence certainty or probability of clinical success. Most importantly, laboratory outcomes do not establish a tolerable tissue exposure, clinical cure, device salvage, antibiotic dose reduction or resistance prevention in patients.

### 4.7 Priorities for future experiments

The next studies should define formulation and final exposure chemistry, include complete matched controls, and use a clinically relevant background regimen. Prevention and established treatment should be planned as separate experiments, with viable burden, retained and released biomass, regrowth and resistance outcomes selected according to intent. Disease-relevant media, surfaces and flow should be chosen explicitly, not added solely to increase apparent model complexity. Biological replication, detection limits and uncertainty should be reported at the strain/comparison level, with a stated interaction model appropriate to the measured endpoint [67].

## 5 Conclusions

NAC can modify antibiotic-associated biofilm outcomes, but the direction and meaning of that modification depend on formulation, pH, medium, hydrodynamics, organism, treatment phase and endpoint. The evidence supports targeted preclinical testing rather than a class-wide claim of synergy. Polymyxin results do not justify clinical dose reduction, and rifampicin experiments must be interpreted against the correct multidrug background. Better controlled comparisons, reproducible searches and independent validation of extraction are necessary before drawing stronger clinical conclusions.

## CRediT authorship contribution statement

**Bakhytzhan Seksenbayev:** Writing – original draft, Methodology, Investigation, Formal analysis, Data curation, Conceptualization. **Yerlan Suleimenov:** Writing – review & editing, Validation, Methodology, Investigation, Formal analysis. **Assylkhan Bekbayev:** Writing – review & editing, Visualization, Validation, Investigation, Data curation. **Nurlan Sadykov:** Writing – review & editing, Validation, Software, Methodology, Formal analysis. **Aziza Omari:** Writing – review & editing, Validation, Supervision, Project administration, Methodology, Conceptualization.

## Ethics approval and consent to participate

Not applicable; this review analyzes published in-vitro research. Consent for publication: Not applicable.

## Registration

The review was not prospectively registered.

## Use of generative AI

Generative AI tools assisted source indexing, preparation of extraction drafts, coding, manuscript preparation and language editing. The verification procedures and reporting limitations are described in Methods. The authors retain responsibility for the accuracy and integrity of the submitted work.

## Funding-related disclosure

This research received no specific grant from any funding agency in the public, commercial, or not-for-profit sectors.

The article publication charge is expected to be covered by Astana Medical University. Astana Medical University had no role in the review design, study selection, data extraction, analysis, interpretation, manuscript preparation, or decision to submit.

## Declaration statement

All authors certify that they have completed the journal's declarations tool. Each author selected the option “I have nothing to declare.”

The authors declare that they have no known financial or personal relationships with other people or organizations that could inappropriately influence or bias the work reported in this manuscript.

Specifically, the authors declare no competing interests related to employment, consultancies, stock ownership, honoraria, paid expert testimony, patent applications or registrations, grants or any other funding, or affiliation with the journal as an Editor or Advisory Board Member.

No author signatures are required for this declaration.

## Data Availability

Data will be made available on request. Mendeley DataN-Acetylcysteine as an Adjunct to Antibiotics against Bacterial Biofilms: A Systematic Review of In Vitro Evidence (Original data)
