# Supplementary material for: N-acetylcysteine as an adjunct to antibiotics against bacterial biofilms: A systematic review of in vitro evidence
Source: Biofilm. 2026 Sep 23;12:100402. doi: 10.1016/j.bioflm.2026.100402 (PMC13634462; doi:10.1016/j.bioflm.2026.100402)
Supplement: Supplementary file 1 [file mmc1.docx]

# Supplementary material

# N-Acetylcysteine as an Adjunct to Antibiotics against Bacterial Biofilms: A Systematic Review of In Vitro Evidence

# S1. Extraction framework and interpretation rules

| **Domain** | **Recorded information** | **Interpretation** |
| --- | --- | --- |
| Intervention | NAC formulation, concentration and carrier; stock and exposure pH; acid-only/pH-matched controls | Stock adjustment, measured treatment pH and matched-pH controls are distinct. Nominal loading is not an eluted exposure. |
| Comparators | Antibiotic regimen without NAC; NAC-alone arm; carrier controls; antibiotic-dose matching | Documented, partial/formulation-specific and not-identified coverage delimit attribution. Unequal-dose and historical comparisons are contextual. |
| Phase | Combination timing during formation (F), after establishment (E), or resistance evolution (R) | Classifications follow actual exposure timing. F + E studies contribute to both phase-component totals but are not independent studies. |
| Intent and endpoint | Prevention/anti-adhesion, viable-cell reduction, matrix/biomass effects, delivery or resistance modulation | Intent is separate from phase. Biomass loss, killing, susceptibility change and formal interaction are not interchangeable. |
| Experimental setting | Growth and exposure media; substrate; static, agitated/mixed, unspecified batch or continuous-flow conditions | Medium replacement or recovery vortexing is not flow. Clinical context is derived from the stated design, not inferred from the species. |
| Synthesis | Study/phase records and 25 focused comparison records; narrative contextual synthesis | No whole-study efficacy voting, cross-class success ranking, pooled effect or formal subgroup interaction test. |
| Source traceability | Full texts of all 55 included reports; identifiers, filenames, page locators and checksums | Full-text availability permits checking but does not resolve missing or inconsistent reporting. |
| Numerical data | Selected reported values, units and uncertainty labels; control sets specific to each assay | No figure digitization, missing-value imputation, or conversion of inconsistent SD/SEM labels into effect-size variances. |
| Search and selection | Recorded search strategies/yields; full-text exclusions and unretrieved reports | Raw exports and exact dates were unavailable. Included-source verification is distinct from verification of the screening record. |
| Analytic status | Exploratory experimental-context stratification; domain-based interpretability appraisal | The review was not prospectively registered. The appraisal is not a validated risk-of-bias score or a formal certainty grading. |

F = combination exposure during initial formation; E = treatment after establishment; R = resistance-evolution purpose. Study/phase records are not independent experiments. A shared study-level outcome summary must not be read as an identical effect in each phase. The focused comparison table in the data workbook resolves selected within-study differences but is not exhaustive arm-level extraction.

“Not reported” describes available source information; “not identified” does not prove that a control was absent. Preparation pH, exposure pH and matched-pH controls are distinct. The source-file inventory includes checksums for traceability. Source PDFs are not redistributed in this package.

# S2. Database strategies and reported yields

The database strategies and recorded yields below relate to searches through August 2026. Exact database-specific execution dates, raw exports and saved search histories were unavailable for verification. The reported PubMed yield of two records could not be reconciled with a search history. Contributions from Google Scholar and citation searching were not separately reconstructible. The strategies and counts should therefore be interpreted with these reproducibility limitations.

## PubMed/MEDLINE — reported yield: 2

(

"Acetylcysteine"[Mesh]

OR acetylcysteine[Title/Abstract]

OR "N-acetylcysteine"[Title/Abstract]

OR "N acetylcysteine"[Title/Abstract]

OR "N-acetyl-L-cysteine"[Title/Abstract]

OR "N acetyl L cysteine"[Title/Abstract]

OR NAC[Title/Abstract]

)

AND

(

"Anti-Bacterial Agents"[Mesh]

OR antibiotic*[Title/Abstract]

OR antibacterial*[Title/Abstract]

OR antimicrobial*[Title/Abstract]

OR antiinfective*[Title/Abstract]

OR "anti-infective"[Title/Abstract]

OR bactericid*[Title/Abstract]

OR bacteriostatic*[Title/Abstract]

OR beta-lactam*[Title/Abstract]

OR betalactam*[Title/Abstract]

OR aminoglycoside*[Title/Abstract]

OR fluoroquinolone*[Title/Abstract]

OR quinolone*[Title/Abstract]

OR macrolide*[Title/Abstract]

OR tetracycline*[Title/Abstract]

OR glycopeptide*[Title/Abstract]

OR lipopeptide*[Title/Abstract]

OR polymyxin*[Title/Abstract]

OR sulfonamide*[Title/Abstract]

OR carbapenem*[Title/Abstract]

OR cephalosporin*[Title/Abstract]

OR penicillin*[Title/Abstract]

OR tigecycline[Title/Abstract]

OR ciprofloxacin[Title/Abstract]

OR levofloxacin[Title/Abstract]

OR amikacin[Title/Abstract]

OR gentamicin[Title/Abstract]

OR tobramycin[Title/Abstract]

OR aztreonam[Title/Abstract]

OR ampicillin[Title/Abstract]

OR doxycycline[Title/Abstract]

OR metronidazole[Title/Abstract]

OR fosfomycin[Title/Abstract]

OR colistin[Title/Abstract]

OR vancomycin[Title/Abstract]

OR linezolid[Title/Abstract]

OR rifampicin[Title/Abstract]

OR rifampin[Title/Abstract]

)

AND

(

"Biofilms"[Mesh]

OR biofilm*[Title/Abstract]

OR bio-film*[Title/Abstract]

OR "sessile bacteria"[Title/Abstract]

OR "sessile bacterial cells"[Title/Abstract]

OR "sessile cells"[Title/Abstract]

OR "biofilm-associated"[Title/Abstract]

OR "biofilm embedded"[Title/Abstract]

OR "biofilm-embedded"[Title/Abstract]

)

## Scopus — reported yield: 358

TITLE-ABS-KEY(

acetylcysteine

OR "N-acetylcysteine"

OR "N acetylcysteine"

OR "N-acetyl-L-cysteine"

OR "N acetyl L cysteine"

OR NAC

)

AND

TITLE-ABS-KEY(

antibiotic*

OR antibacterial*

OR antimicrobial*

OR antiinfective*

OR "anti-infective*"

OR bactericid*

OR bacteriostatic*

OR beta-lactam*

OR betalactam*

OR aminoglycoside*

OR fluoroquinolone*

OR quinolone*

OR macrolide*

OR tetracycline*

OR glycopeptide*

OR lipopeptide*

OR polymyxin*

OR sulfonamide*

OR carbapenem*

OR cephalosporin*

OR penicillin*

OR tigecycline

OR ciprofloxacin

OR levofloxacin

OR amikacin

OR gentamicin

OR tobramycin

OR aztreonam

OR ampicillin

OR doxycycline

OR metronidazole

OR fosfomycin

OR colistin

OR vancomycin

OR linezolid

OR rifampicin

OR rifampin

)

AND

TITLE-ABS-KEY(

biofilm*

OR "bio-film*"

OR "sessile bacteria"

OR "sessile bacterial cells"

OR "sessile cells"

OR "biofilm-associated"

OR "biofilm embedded"

OR "biofilm-embedded"

)

## EMBASE Ovid — reported yield: 368

(

exp acetylcysteine/

or

(acetylcysteine or N-acetylcysteine or N acetylcysteine or

N-acetyl-L-cysteine or N acetyl L cysteine or NAC).ti,ab,kw.

)

and

(

exp antibiotic agent/

or

(antibiotic* or antibacterial* or antimicrobial* or antiinfective* or

anti-infective* or bactericid* or bacteriostatic* or beta-lactam* or

betalactam* or aminoglycoside* or fluoroquinolone* or quinolone* or

macrolide* or tetracycline* or glycopeptide* or lipopeptide* or

polymyxin* or sulfonamide* or carbapenem* or cephalosporin* or

penicillin* or tigecycline or ciprofloxacin or levofloxacin or

amikacin or gentamicin or tobramycin or aztreonam or ampicillin or

doxycycline or metronidazole or fosfomycin or colistin or vancomycin or

linezolid or rifampicin or rifampin).ti,ab,kw.

)

and

(

exp biofilm/

or

(biofilm* or bio-film* or sessile bacteria or sessile bacterial cells or

sessile cells or biofilm-associated or biofilm embedded or

biofilm-embedded).ti,ab,kw.

)

## WoS — reported yield: 293

TS=(

acetylcysteine

OR "N-acetylcysteine"

OR "N acetylcysteine"

OR "N-acetyl-L-cysteine"

OR "N acetyl L cysteine"

OR NAC

)

AND TS=(

antibiotic*

OR antibacterial*

OR antimicrobial*

OR antiinfective*

OR "anti-infective*"

OR bactericid*

OR bacteriostatic*

OR beta-lactam*

OR betalactam*

OR aminoglycoside*

OR fluoroquinolone*

OR quinolone*

OR macrolide*

OR tetracycline*

OR glycopeptide*

OR lipopeptide*

OR polymyxin*

OR sulfonamide*

OR carbapenem*

OR cephalosporin*

OR penicillin*

OR tigecycline

OR ciprofloxacin

OR levofloxacin

OR amikacin

OR gentamicin

OR tobramycin

OR aztreonam

OR ampicillin

OR doxycycline

OR metronidazole

OR fosfomycin

OR colistin

OR vancomycin

OR linezolid

OR rifampicin

OR rifampin

)

AND TS=(

biofilm*

OR "bio-film*"

OR "sessile bacteria"

OR "sessile bacterial cells"

OR "sessile cells"

OR "biofilm-associated"

OR "biofilm embedded"

OR "biofilm-embedded"

)

## All results — reported yield: 1021

# S3. Full-text exclusions and reports not retrieved

## Nostro, A., et al. (2026) | record 343

Design of polymeric nanoantibiotics: N-acetylcysteine-functionalized PLA-PEG nanoparticles for linezolid and pentamidine delivery.

Reason: No matched antibiotic-without-NAC formulation comparator

Verification basis: Screening record; full report unavailable for independent confirmation.

## Ahmad, M., et al. (2025) | record 19

Targeting Antimicrobial Resistance in Salmonella Enteritidis: Synergistic Action of N-acetyl cysteine with Florfenicol and CRISPR-Cas System Involvement.

Reason: No separable experimental biofilm-specific NAC–antibiotic outcome; reported methods/outcomes concern planktonic MIC/MBC/MPC, interaction, time-kill and gene-expression assays.

Verification basis: Full report.

## Recinella, L., et al. (2025) | record 402

A New Bromelain-Based Polyenzymatic Complex Plus N-Acetylcysteine: A Promising Approach for the Treatment of Urinary Tract Infections.

Reason: Co-intervention confounding; NAC-specific effect not isolatable

Verification basis: Screening record; full report unavailable for independent confirmation.

## Yaseen, K., et al. (2025) | record 489

Surface engineering of biomedical catheters using N-acetyl cysteine functionalized carboxymethyl chitosan nanosystems to combat biofouling and device-associated infections.

Reason: No matched antibiotic-only surface/formulation comparator

Verification basis: Screening record; full report unavailable for independent confirmation.

## Aljihani, S. A., et al. (2020) | record 37

Enhancing azithromycin antibacterial activity by encapsulation in liposomes/liposomal-N-acetylcysteine formulations against resistant clinical strains of Escherichia coli.

Reason: Unmatched liposomal formulation comparator

Verification basis: Full report.

## Manoharan, A., et al. (2020) | record 305

The effect of N-acetylcysteine in a combined antibiofilm treatment against antibiotic-resistant Staphylococcus aureus.

Reason: Co-intervention confounding; no antibiotic+enzyme comparator without NAC

Verification basis: Screening record; full report unavailable for independent confirmation.

## Lababidi, N., et al. (2019) | record 271

Spray-drying of inhalable, multifunctional formulations for the treatment of biofilms formed in cystic fibrosis.

Reason: Unmatched spray-dried formulation comparator

Verification basis: Full report.

## de Sousa, J. K. T., et al. (2019) | record 145

In vitro activity of antimicrobial-impregnated catheters against biofilms formed by KPC-producing Klebsiella pneumoniae.

Reason: No matched antibiotic-only impregnated catheter comparator

Verification basis: Full report.

## Aiassa, V., et al. (2016) | record 23

Enhanced inhibition of bacterial biofilm formation and reduced leukocyte toxicity by chloramphenicol:beta-cyclodextrin:N-acetylcysteine complex.

Reason: No matched chloramphenicol-cyclodextrin comparator without NAC

Verification basis: Screening record; full report unavailable for independent confirmation.

## Mansouri, M. D., et al. (2013) | record 312

In vitro activity and durability of a combination of an antibiofilm and an antibiotic against vascular catheter colonization.

Reason: Preventive impregnated-device model with no matched antibiotic-only catheter

Verification basis: Screening record; full report unavailable for independent confirmation.

Ahmad et al. (2025) was assessed against the primary report (doi:10.29261/pakvetj/2025.212); the exclusion concerns the absence of a separable biofilm-specific NAC–antibiotic comparison, not a restriction against formation studies. For exclusions without an accessible full report, the recorded reason could not be independently confirmed.

## Reports not retrieved (n = 5)

Bartolilla, A., et al. (2026) — From ascorbic acid to N-acetylcysteine: self-assembled clarithromycin liquid crystals as a new generation of carrier-free antibiotics. Eur J Pharm Biopharm 222: 115026.

Xu, M., et al. (2025) — Tailored multilayer nanoparticles against resistant P. aeruginosa by disrupting the thickened mucus, dense biofilm and hyperinflammation. J Control Release 378: 588-604.

Mohamed, H., et al. (2023) — Effect of chitosan or N-Acetyl cysteine combinations with some antibiotics on biofilm formation on intrauterine devices. Bulletin of Pharmaceutical Sciences Assiut University 0(0): 0-0.

Temel, A. and B. Erac (2022) — Investigating Biofilm Formation and Antibiofilm Activity Using Real Time Cell Analysis Method in Carbapenem Resistant Acinetobacter baumannii Strains. Curr Microbiol 79(9): 256.

Yilmaz, F. N., et al. (2022) — Impact of N-Acetylcysteine and Antibiotics Against Single and Dual Species Biofilms of Pseudomonas aeruginosa and Achromobacter xylosoxidans. Curr Microbiol 80(1): 5.

# S4. Study-level extraction and source notes

The study inventory contains 55 included reports, each assessed in full text. Stable study identifiers link the extraction, phase map, focused comparisons and source inventory. Page locators refer to the page sequence of the source file unless a printed page is explicitly stated. Missing information and inconsistent source descriptions are retained in the notes rather than resolved by assumption.

## S01 | Marchese, A., et al. (2003)

System / phase / intent: 4 slime-producing uropathogenic Escherichia coli strains; 96-well polystyrene biofilm | 5–6-h initial and 48-h mature biofilms; 24-h treatment. Phase E. Viability reduction + biomass disruption. Context: Urinary / uropathogenic isolates.

Intervention and comparison: Fosfomycin | Free NAC concentration series. Fosfomycin alone vs fosfomycin + NAC. Formulation: Free NAC. NAC-alone: Present.

Exposure: Stock pH — Not reported. Exposure pH — Not reported for the relevant biofilm exposure. pH control — Not identified. Growth medium — TSB. Treatment medium — MHB. Hydrodynamics — Batch; agitation not reported.

Outcome / interpretation: Residual biofilm/slime biomass; sessile-cell viability. No explicit biofilm interaction threshold identified. Fosfomycin plus NAC reduced viable burden/biomass in early and mature E. coli biofilms; NAC-alone formation experiments are not counted as combination prevention evidence.

Verification note: 5–6-h biofilms are early established, not prophylaxis. Broth replacement is not continuous flow.

Source: Full text verified; Marchese.pdf; PDF pages 1–3.

## S02 | Aslam, S., et al. (2007)

System / phase / intent: MRSA, MSSA, MRSE, VRE, K. pneumoniae and A. baumannii clinical catheter isolates; Vascular catheter biofilm | 24-h biofilm followed by 4- or 12-h treatment. Phase E. Viable-cell killing / catheter decontamination. Context: Vascular catheter.

Intervention and comparison: Tigecycline | Free NAC 80 mg/mL. Tigecycline alone vs tigecycline + NAC. Formulation: Free NAC. NAC-alone: Present.

Exposure: Stock pH — Not reported. Exposure pH — Not reported for the relevant biofilm exposure. pH control — Not identified. Growth medium — TSB. Treatment medium — Saline-based catheter treatment solutions. Hydrodynamics — Batch; agitation not reported.

Outcome / interpretation: CFU/cm; eradication; synergy assessment. Study-defined viable-count synergy comparison. Tigecycline addition contrasts favored the combination for several catheter-associated organisms; NAC alone was active against some isolates.

Source: Full text verified; Aslam.pdf; PDF pages 1–2.

## S03 | El-Feky, M. A., et al. (2009)

System / phase / intent: 12 clinical isolates: 2 each S. aureus, S. epidermidis, E. coli, K. pneumoniae, P. aeruginosa, P. vulgaris; JJ ureteral-stent segments; static adherence assay | Both formation inhibition and 24-h preformed biofilm eradication; 24-h treatment. Phase F;E. Anti-colonization + viable-cell reduction. Context: Urinary device.

Intervention and comparison: Ciprofloxacin | Free NAC 2 or 4 mg/mL. Ciprofloxacin alone vs ciprofloxacin + NAC. Formulation: Free NAC. NAC-alone: Present.

Exposure: Stock pH — Not reported. Exposure pH — Not reported for the relevant biofilm exposure. pH control — Not identified. Growth medium — TSB. Treatment medium — Agent-containing assay solutions; saline control. Hydrodynamics — Static.

Outcome / interpretation: Viable CFU; % reduction/eradication; SEM. Incremental effect; no formal biofilm interaction model identified. Ciprofloxacin plus NAC reduced adherent CFU during formation and after treatment of established stent biofilms.

Verification note: PBS pH 7.2 is a wash/fixation detail, not evidence of NAC neutralization.

Source: Full text verified; El-Feky.pdf; PDF pages 2–4.

## S04 | Venkatesh, M., et al. (2009)

System / phase / intent: Staphylococcus epidermidis ATCC 55133, H100 and S101; 96-well preformed biofilm; XTT + confocal | 24-h preformed biofilm before combination exposure. Phase E. Metabolic inhibition / biofilm viability. Context: Catheter-lock model.

Intervention and comparison: Vancomycin; nafcillin | Free NAC. Each antibiotic alone vs antibiotic + NAC. Formulation: Free NAC. NAC-alone: Present.

Exposure: Stock pH — Adjusted to 7. Exposure pH — RPMI 1640 specified at pH 7; post-addition pH not separately documented. pH control — Not identified. Growth medium — RPMI 1640. Treatment medium — RPMI 1640. Hydrodynamics — Batch; agitation not reported.

Outcome / interpretation: MBEC50/XTT; Combination Index at ED50/ED75/ED90; biomass/thickness. Combination index at ED50/75/90; biofilm endpoint. Antibiotic–NAC interactions were evaluated in established single- and mixed-species biofilms; only bacterial antibiotic contrasts contribute to this review.

Verification note: Fungal-only and non-antibiotic combinations are outside the synthesis.

Source: Full text verified; Venkatesh.pdf; PDF pages 2–3.

## S05 | Zhao et al. (2010)

System / phase / intent: Pseudomonas aeruginosa PAO1 + 20 respiratory clinical isolates; Silicone-sheet mature biofilm; CLSM/COMSTAT | 6-day mature biofilm; combination treatment over 24 h. Phase E. Metabolic viability + matrix disruption. Context: Respiratory isolates; CF not assumed.

Intervention and comparison: Ciprofloxacin | Free NAC concentration series. Same ciprofloxacin concentration without vs with NAC. Formulation: Free NAC. NAC-alone: Present.

Exposure: Stock pH — Adjusted to 7.2. Exposure pH — Not reported for the relevant biofilm exposure. pH control — Not identified. Growth medium — TSB; separate AP medium for EPS assays. Treatment medium — Assay-specific; final combination exposure medium not unambiguously separated. Hydrodynamics — Batch; agitation not reported.

Outcome / interpretation: MTT-based estimate of viable burden; CLSM architecture; extracellular polysaccharide measurements. Checkerboard-like concentrations; no formal biofilm synergy threshold identified. Ciprofloxacin plus NAC reduced the MTT-derived viable-cell estimate and altered mature P. aeruginosa biofilm structure.

Verification note: MTT was calibrated against viable counts; it is not direct plating of each treated biofilm. Mean ± SD was reported. The NAC stock was adjusted to pH 7.2; stock adjustment does not establish final exposure pH.

Source: Full text verified; Zhao.pdf; PDF pages 5–7.

## S06 | del Prado, G., et al. (2010)

System / phase / intent: Streptococcus pneumoniae; Microplate pneumococcal biofilm | 16-h biofilm followed by ~5-h exposure. Phase E. Viable-cell reduction. Context: Pneumococcal respiratory infection.

Intervention and comparison: Amoxicillin; erythromycin; levofloxacin | Free NAC. Each antibiotic alone vs same antibiotic + NAC. Formulation: Free NAC. NAC-alone: Present.

Exposure: Stock pH — Not reported. Exposure pH — Not reported for the relevant biofilm exposure. pH control — Not identified. Growth medium — Pneumococcal culture medium as described in source. Treatment medium — Pneumococcal assay medium; see source. Hydrodynamics — Batch; agitation not reported.

Outcome / interpretation: CFU/log change; biofilm response. Incremental effect; no common interaction model. NAC produced limited, antibiotic- and strain-dependent changes in established pneumococcal biofilms.

Source: Full text verified; del Prado.pdf; PDF pages 1–3.

## S07 | Abbas, H. A., et al. (2012)

System / phase / intent: Pseudomonas aeruginosa; 5 clinical ICU isolates; Microplate established-biofilm regrowth model | ~24-h preformed biofilm; subsequent 18–20-h antimicrobial exposure/recovery. Phase E. Suppression of biofilm-derived regrowth. Context: P. aeruginosa; no site-specific model.

Intervention and comparison: Ciprofloxacin; cefoperazone; tobramycin; amikacin; imipenem | Free NAC at sub-MRC concentrations. Each antibiotic alone vs same antibiotic + NAC. Formulation: Free NAC. NAC-alone: Present.

Exposure: Stock pH — Not reported. Exposure pH — Not reported for the relevant biofilm exposure. pH control — Not identified. Growth medium — TSB. Treatment medium — MHB treatment; TSB regrowth. Hydrodynamics — Batch; agitation not reported.

Outcome / interpretation: Minimum regrowth concentration (MRC); antibiotic concentration-sparing effect. Regrowth concentration shifts, not a formal synergy model. NAC reduced required antibiotic concentrations in a preformed-biofilm/regrowth assay; this is not an eradication endpoint confirmed by direct viable counts.

Source: Full text verified; Abbas.pdf; PDF pages 2–3.

## S08 | Abd El-Aziz, A. A., et al. (2012)

System / phase / intent: Pseudomonas aeruginosa Ps22 clinical isolate; Microtiter plate and urinary-catheter segment biofilm models | Both formation inhibition and 24-h preformed biofilm disruption; 24-h treatment. Phase F;E. Anti-colonization + viable-cell reduction. Context: Urinary catheter.

Intervention and comparison: Ciprofloxacin | Free NAC (0.5 and 2 mg/mL in key arms). Ciprofloxacin alone vs ciprofloxacin + NAC. Formulation: Free NAC. NAC-alone: Present.

Exposure: Stock pH — Not reported. Exposure pH — Not reported for the relevant biofilm exposure. pH control — Not identified. Growth medium — MHB. Treatment medium — MHB for formation; catheter treatment solutions. Hydrodynamics — Static.

Outcome / interpretation: Biofilm formation reduction; preformed-biofilm eradication; catheter CFU. Incremental biofilm contrast; planktonic susceptibility separate. Ciprofloxacin plus NAC reduced formation and catheter-associated burden in selected isolates.

Verification note: PBS pH 7.2 is a wash/fixation buffer; the MBC neutralizer is a recovery step, not NAC stock buffering.

Source: Full text verified; A. A. Abd El-Aziz.pdf; PDF pages 2–4.

## S09 | Leite et al. (2013a)

System / phase / intent: Staphylococcus epidermidis strains 9142 and 1457; 96-well preformed biofilm | 24-h preformed biofilm followed by 24-h treatment. Phase E. Viable-cell reduction + biomass/metabolic outcomes. Context: Staphylococcal device-relevant isolates.

Intervention and comparison: Linezolid | Free NAC at MIC and 10×MIC. Linezolid alone vs linezolid + NAC. Formulation: Free NAC. NAC-alone: Present.

Exposure: Stock pH — Not reported. Exposure pH — Not reported for the relevant biofilm exposure. pH control — Not identified. Growth medium — TSB + 0.25% glucose. Treatment medium — Fresh TSB. Hydrodynamics — Agitated batch.

Outcome / interpretation: CFU; XTT metabolic activity; crystal-violet biomass. Viable-count interaction interpretation; endpoint-specific. Linezolid plus NAC reduced established S. epidermidis biofilms, but high NAC concentrations were independently active.

Verification note: Orbital shaking at 130 rpm was reported.

Source: Full text verified; Leite, Bruna.pdf; PDF pages 2–4.

## S10 | Leite et al. (2013b)

System / phase / intent: 5 S. epidermidis + 5 S. aureus clinical isolates; 96-well agitated biofilm | 24-h preformed biofilm followed by 24-h treatment. Phase E. Viable-cell reduction. Context: Staphylococcal device-relevant isolates.

Intervention and comparison: Vancomycin | Free NAC at MIC and 10×MIC. Vancomycin alone vs vancomycin + NAC. Formulation: Free NAC. NAC-alone: Present.

Exposure: Stock pH — Not reported. Exposure pH — Not reported for the relevant biofilm exposure. pH control — Not identified. Growth medium — TSB + 0.25% glucose. Treatment medium — Fresh nutrient medium (TSB protocol). Hydrodynamics — Agitated batch.

Outcome / interpretation: CFU/mL; biofilm viability. Incremental effect; NAC-alone activity substantial. High-concentration NAC alone reduced staphylococcal viable counts; the incremental effect of vancomycin must be distinguished from independent NAC killing.

Verification note: Orbital shaking 130 rpm, not static.

Source: Full text verified; Leite.pdf; PDF pages 2–4.

## S11 | Leite et al. (2013c)

System / phase / intent: Staphylococcus epidermidis strains 9142 and 1457; 96-well tissue-culture plate biofilm | 24-h preformed biofilm followed by 24-h treatment. Phase E. Viable-cell reduction + matrix/metabolic outcomes. Context: Staphylococcal device-relevant isolates.

Intervention and comparison: Rifampicin | Free NAC 4 or 40 mg/mL. Rifampicin alone vs rifampicin + NAC. Formulation: Free NAC. NAC-alone: Present.

Exposure: Stock pH — Not reported. Exposure pH — Not reported for the relevant biofilm exposure. pH control — Not identified. Growth medium — TSB + 0.25% glucose. Treatment medium — Fresh TSB. Hydrodynamics — Agitated batch.

Outcome / interpretation: CFU; XTT metabolic activity; crystal-violet biomass. Incremental effect; no clinical-regimen comparator. Rifampicin alone was active in the selected biofilms, leaving limited incremental change in several conditions; this does not compare NAC against clinical rifampicin-containing regimens.

Verification note: PBS/extraction-buffer pH 7.0 belongs to EPS recovery, not NAC exposure. 130 rpm.

Source: Full text verified; B. Leite, F. Gomes.pdf; PDF pages 2–4.

## S12 | Lea, J., et al. (2014)

System / phase / intent: Pseudomonas aeruginosa clinical isolates from chronic suppurative otitis media; Peg-based biofilm susceptibility/regrowth assay | ~5-h early established biofilm before challenge. Phase E. Biofilm-derived regrowth inhibition. Context: Chronic suppurative otitis media.

Intervention and comparison: Ciprofloxacin; Ciprodex | Free NAC. Ciprofloxacin or Ciprodex alone vs same treatment + NAC. Formulation: Free NAC + ciprofloxacin or Ciprodex. NAC-alone: Present.

Exposure: Stock pH — Not reported. Exposure pH — Not reported for the relevant biofilm exposure. pH control — Not identified. Growth medium — TSB. Treatment medium — Treatment solutions; CAMHB recovery. Hydrodynamics — Agitated batch.

Outcome / interpretation: Optical-density/regrowth resistance endpoint. Susceptibility/regrowth categories; not a formal synergy model. Response depended on NAC concentration; 0.25% NAC yielded inferior sessile susceptibility in some comparisons, whereas higher concentrations were more active.

Verification note: Peg growth 5 h at 110 rpm; early established biofilm. Ciprodex contains dexamethasone, which must be retained in its matched comparator.

Source: Full text verified; Lea.pdf; PDF pages 2–5.

## S13 | Moon, J. H., et al. (2015)

System / phase / intent: Prevotella intermedia ATCC 49046; 96-well pre-established biofilm; LIVE/DEAD CLSM | 24-h pre-established biofilm followed by 24-h treatment. Phase E. Biofilm viability / antibacterial interaction. Context: Oral / periodontal.

Intervention and comparison: Ampicillin; ciprofloxacin; tetracycline; metronidazole | Free NAC 0.75–3 mg/mL. Each antibiotic alone vs same antibiotic + NAC. Formulation: Free intrinsic-pH NAC. NAC-alone: Present.

Exposure: Stock pH — Unneutralized aqueous stock. Exposure pH — Dose-dependent culture acidification measured. pH control — Separate acidic-medium controls; not a complete pH-matched factorial biofilm design. Growth medium — Brucella-based B-HK medium. Treatment medium — B-HK medium. Hydrodynamics — Batch; agitation not reported.

Outcome / interpretation: Biofilm viability; CLSM; planktonic FICI. Planktonic FICI; biofilm viability assessed separately. NAC reduced ampicillin, ciprofloxacin and tetracycline activity in selected conditions; metronidazole was unaffected and is not classified as antagonistic.

Verification note: Acidic-medium controls do not eliminate every exposure-pH confound.

Source: Full text verified; Moon.pdf; PDF pages 2–4, 7.

## S14 | Espinosa, I., et al. (2016)

System / phase / intent: Streptococcus suis; Microtiter formation assay plus preformed biofilms aged 4, 8 and 24 h. Phase F;E. Formation inhibition + early biofilm biomass response. Context: Veterinary / Streptococcus suis.

Intervention and comparison: Penicillin G | Free NAC. Penicillin G alone vs penicillin G + NAC. Formulation: Free NAC. NAC-alone: Present.

Exposure: Stock pH — Not reported. Exposure pH — Not reported for the relevant biofilm exposure. pH control — Not identified. Growth medium — Minimal medium (MM). Treatment medium — MM. Hydrodynamics — Batch; agitation not reported.

Outcome / interpretation: Sessile/planktonic inhibition; biofilm development/disruption. Inhibition shifts; no explicit biofilm interaction threshold identified. Penicillin G plus NAC had assay-age-dependent effects; early effects did not demonstrate sustained eradication of older biofilms.

Verification note: Endpoint wording in source mixes growth inhibition and biofilm development; do not relabel all outcomes as killing.

Source: Full text verified; Espinosa.pdf; PDF pages 1–3.

## S15 | Molina-Manso, D., et al. (2016)

System / phase / intent: Clinical S. aureus and S. epidermidis from prosthetic joint infections; Calgary/MBEC device assay | Established biofilm susceptibility / MBEC. Phase E. Biofilm-derived regrowth / MBEC. Context: Prosthetic joint infection isolates.

Intervention and comparison: Multiple: rifampin, vancomycin, tigecycline, clindamycin, cotrimoxazole, ciprofloxacin, cloxacillin, daptomycin, fosfomycin, linezolid | NAC 1024 mg/L. Antibiotic+NAC vs antibiotic-alone MBEC generated using identical methodology. Formulation: Free NAC. NAC-alone: Not identified for the matched biofilm contrast.

Exposure: Stock pH — Not reported. Exposure pH — Not reported for the relevant biofilm exposure. pH control — Not identified. Growth medium — MBEC/Calgary protocol; source refers to earlier method. Treatment medium — MBEC susceptibility assay; historical antibiotic comparisons. Hydrodynamics — Batch; agitation not reported.

Outcome / interpretation: MBEC; strain-level susceptibility shifts. Historical antibiotic-alone benchmark; no concurrent interaction estimate. No consistent improvement for S. aureus; S. epidermidis effects varied by strain and antibiotic, including increased MBECs.

Verification note: Historical/non-concurrent comparators are retained as contextual evidence only, not confirmatory potentiation.

Source: Full text verified; Molina-Manso.pdf; PDF pages 1–2.

## S16 | Munoz-Egea, M. C., et al. (2016)

System / phase / intent: Rapidly growing mycobacteria: M. abscessus, M. chelonae, M. fortuitum, M. smegmatis; Calgary 96-h biofilm + 48-h treatment; microscopy with exposure after 24, 48 and 72 h. Phase E. Established-biofilm structural/viability response and MBEC. Context: Rapidly growing mycobacteria / device-relevant.

Intervention and comparison: Ciprofloxacin; amikacin; clarithromycin | Free NAC. Antibiotic+NAC vs antibiotic-alone data generated by identical methodology. Formulation: Free NAC. NAC-alone: Not identified for every historical antibiotic contrast.

Exposure: Stock pH — Not reported. Exposure pH — Not reported for the relevant biofilm exposure. pH control — Not identified. Growth medium — Middlebrook 7H9 after initial PBS adhesion (microscopy); Calgary assay as cited. Treatment medium — Middlebrook 7H9 (microscopy); Calgary assay medium not restated. Hydrodynamics — Agitated batch.

Outcome / interpretation: MBEC; CLSM biofilm thickness/covered surface/live-dead. Some antibiotic-alone comparisons historical. NAC-associated changes depended on species, antibiotic and biofilm age; historical comparisons limit attribution.

Verification note: Antibiotic/NAC exposures at 24, 48 and 72 h; 96-h Calgary biofilms exposed for 48 h. Some antibiotic-only MBEC and ciprofloxacin microscopy comparisons use historical results; not independent confirmation of potentiation.

Source: Full text verified; Munoz-Egea.pdf; PDF pages 2–4.

## S17 | Onger, M. E., et al. (2016)

System / phase / intent: Pseudomonas aeruginosa PAO1 (ATCC 15692); NAC/ciprofloxacin premixed into bone cement before inoculation; 48-h development on cement. Formation/prevention by exposure timing; source also uses preformed/eradication language without a separate delayed-treatment protocol. Phase F. Prevention/anti-adhesion on drug-loaded cement; viable adherent-burden reduction and SEM. Source also frames eradication; independent established-treatment evidence is not demonstrated by its sequence.. Context: Orthopaedic bone-cement / periprosthetic joint infection model.

Intervention and comparison: Per 40 g cement: ciprofloxacin 2 g with or without NAC 6 mg; released concentrations not measured. Cement + ciprofloxacin 2 g versus same cement + ciprofloxacin 2 g + NAC 6 mg; blank cement and NAC-only cement controls. Formulation: NAC 6 mg (Merck Millipore) incorporated with ciprofloxacin 2 g per 40 g bone cement; no elution concentration characterized. NAC-alone: Present: NAC-only cement 6 mg per 40 g, alongside ciprofloxacin-only, combination and blank cement.

Exposure: Stock pH — Not applicable as a free-drug stock; cement loading reported; preparation pH not reported. Exposure pH — Not reported for the cement/broth biofilm exposure. pH control — No acid-only or pH-matched biofilm control reported. Growth medium — Tryptic soy broth (TSB) supplemented with 0.5% glucose; 48-h inoculation. Treatment medium — TSB + 0.5% glucose during colonization of preloaded cement; saline used after incubation for washing/recovery, not as a separate treatment. Hydrodynamics — Batch incubation on cement; agitation during growth/exposure not reported. Three-minute vortexing is recovery, not flow..

Outcome / interpretation: Recovered adherent CFU; SEM and semiquantitative bacterial score; source SD/SEM and unit-label inconsistencies retained. Four-arm cement comparison; no formal synergy model. Table I shows a lower combination count; Results omit an explicit ciprofloxacin-only versus combination pairwise statistic.. Table I reports mean count entries 4 (ciprofloxacin-only) versus 0.01 (combination), on the same author-labeled scale. The source mixes SD/SEM labels and describes preformed biofilms despite drug incorporation before inoculation. This supports a formulation-specific reduction in recovered colonization, not proven complete eradication or free-drug synergy.

Verification note: NAC loading was reported as 6 mg per 40 g cement; loading does not establish a dissolved exposure concentration. Table I combines CFU 10^8 and SEM in its header, whereas the column heading and statistical methods specify SD. The stated SEM score categories and reported means are also not readily reconcilable. No standardized numerical effect or variance was derived. Drug-loading chronology supports a formation/prevention classification, with the source ambiguity retained.

Source: Full text verified; Onger(2).pdf; PDF pages 2 (loading, medium, sequence); 3 (Table I, statistics, SEM); 4 (Table II and limitations).

## S18 | Belfield, K., et al. (2017)

System / phase / intent: Clinical Staphylococcus aureus and Pseudomonas aeruginosa; Silicone-disc mature biofilm | 5-day mature biofilm; 7- or 14-day treatment. Phase E. Viable-cell reduction / mature device treatment. Context: Silicone device / catheter or implant model.

Intervention and comparison: S. aureus: rifampicin+clindamycin; P. aeruginosa: gentamicin+ciprofloxacin | Free NAC added to the same antibiotic regimens. Same dual-antibiotic regimen without NAC. Formulation: Free neutralized NAC. NAC-alone: Present.

Exposure: Stock pH — Neutralized with NaOH; target pH not specified. Exposure pH — Not reported for the relevant biofilm exposure. pH control — Not identified. Growth medium — TSB; 5-day maturation. Treatment medium — TSB-based treatment protocol. Hydrodynamics — Static maturation; initial attachment includes shaking.

Outcome / interpretation: Viable counts / eradication on silicone biofilm. Matched multi-antibiotic regimen ± NAC. Neutralized NAC added to organism-specific antibiotic regimens did not uniformly improve mature silicone-biofilm eradication.

Verification note: Staphylococcal comparison includes rifampicin + clindamycin; do not classify as rifampicin monotherapy.

Source: Full text verified; Belfield.pdf; PDF pages 2–4.

## S19 | Gocer, H., et al. (2017)

System / phase / intent: Staphylococcus aureus ATCC 25923; Drug-loaded bone cement | Formation/prevention model; drugs incorporated before inoculation; 48-h biofilm development. Phase F. Prevention of cement colonization. Context: Orthopaedic device.

Intervention and comparison: Teicoplanin | NAC incorporated into bone cement (6 g/40 g cement). Cement+teicoplanin vs identical cement+teicoplanin+NAC. Formulation: NAC in teicoplanin-loaded bone cement. NAC-alone: Present.

Exposure: Stock pH — Not reported. Exposure pH — Not reported for the relevant biofilm exposure. pH control — Not identified. Growth medium — TSB + 0.5% glucose. Treatment medium — Same colonization broth; drugs preloaded in cement. Hydrodynamics — Batch; agitation not reported.

Outcome / interpretation: CFU; SEM bacterial/biofilm counts. Incremental effect; no formal interaction model. Teicoplanin–NAC cement reduced colonization more than singly loaded cement; drugs were present before inoculation.

Verification note: Formation/prevention model, despite some wording about preformed biofilms in the report.

Source: Full text verified; Gocer.pdf; PDF pages 2–4.

## S20 | Feng, J., et al. (2018)

System / phase / intent: Acinetobacter baumannii clinical isolates; Static mature-biofilm viable-count assay | 72-h preformed biofilm followed by 24-h treatment. Phase E. Viable-cell reduction. Context: A. baumannii respiratory clinical isolates.

Intervention and comparison: Tigecycline | Free NAC concentration matrix. Tigecycline at matched concentrations with NAC=0 vs +NAC. Formulation: Free NAC. NAC-alone: Present.

Exposure: Stock pH — Not reported. Exposure pH — Not reported for the relevant biofilm exposure. pH control — Not identified. Growth medium — Study broth culture. Treatment medium — Fresh broth for 24-h biofilm exposure. Hydrodynamics — Static.

Outcome / interpretation: Log CFU/cm²; planktonic FICI; SEM. Planktonic FICI separate from biofilm CFU comparison. Tigecycline plus NAC reduced viable burden in established A. baumannii biofilms; NAC-only prevention/disruption experiments are not combination prevention evidence.

Source: Full text verified; Feng.pdf; PDF pages 2–4.

## S21 | Pollini, S., et al. (2018)

System / phase / intent: Acinetobacter baumannii; colistin-susceptible and colistin-resistant strains; Nunc TSP lid/peg biofilm | 7-day preformed biofilm followed by 24-h treatment. Phase E. Viable-cell reduction. Context: A. baumannii; resistant and susceptible backgrounds.

Intervention and comparison: Colistin | Free NAC. Colistin alone vs colistin + NAC. Formulation: Free neutralized NAC. NAC-alone: Present.

Exposure: Stock pH — Adjusted to 6.5–6.8 with NaOH. Exposure pH — Not reported for the relevant biofilm exposure. pH control — Not identified. Growth medium — CAMHB, refreshed daily. Treatment medium — CAMHB. Hydrodynamics — Static.

Outcome / interpretation: CFU/peg; antibiofilm synergy; planktonic checkerboard/time-kill (secondary). Planktonic FICI/time-kill plus separate biofilm CFU contrasts. Colistin plus NAC reduced viable burden in several preformed biofilms, but concentration and strain effects and regrowth in other assays prohibit a uniform susceptibility-restoration claim.

Verification note: Stock pH adjustment was reported; concentrated broth prevented dilution at high NAC concentrations. Final exposure pH was not separately reported.

Source: Full text verified; Pollini 2018.pdf; PDF pages 2–3, 6.

## S22 | Ciacci, N., et al. (2019)

System / phase / intent: Stenotrophomonas maltophilia; 18 clinical isolates; Peg-based biofilm susceptibility model | 24-h preformed biofilm followed by 24-h treatment. Phase E. Viable-cell reduction. Context: S. maltophilia / respiratory-relevant isolates.

Intervention and comparison: Colistin | Free NAC. Colistin alone vs colistin + NAC. Formulation: Free neutralized NAC. NAC-alone: Present.

Exposure: Stock pH — Adjusted to 6.5–6.8 with NaOH. Exposure pH — Not reported for the relevant biofilm exposure. pH control — Not identified. Growth medium — CAMHB. Treatment medium — CAMHB. Hydrodynamics — Static.

Outcome / interpretation: CFU/peg; antibiofilm interaction/synergy. Planktonic checkerboard plus separate biofilm CFU contrasts. Colistin–NAC combinations showed increased activity against selected established S. maltophilia biofilms; this is organism- and exposure-specific.

Verification note: Broth concentration preserved when adding NAC stock.

Source: Full text verified; Ciacci.pdf; PDF pages 10–11.

## S23 | Kuruoglu, T., et al. (2019)

System / phase / intent: Methicillin-susceptible Staphylococcus aureus (MSSA); Ventriculoperitoneal shunt segments | ~48-h preformed biofilm. Phase E. Device-associated viable-cell reduction. Context: Neurosurgical shunt.

Intervention and comparison: Daptomycin; linezolid | Free NAC. Each antibiotic alone vs same antibiotic + NAC. Formulation: Free NAC applied to shunt segments. NAC-alone: Present.

Exposure: Stock pH — Not reported. Exposure pH — Not reported for the relevant biofilm exposure. pH control — Not identified. Growth medium — TSB + 0.5% glucose. Treatment medium — Antibiotic/NAC treatment solutions. Hydrodynamics — Batch; agitation not reported.

Outcome / interpretation: Biofilm CFU/bacterial counts. Incremental effect; no formal biofilm interaction model identified. Daptomycin–NAC and linezolid–NAC did not respond identically in established S. aureus shunt biofilms.

Verification note: Use post-formation timing from Methods, not a general prevention statement in the Introduction.

Source: Full text verified; Kuruoglu 2018.pdf; PDF pages 2–3.

## S24 | Rastegar Khosravi, M., et al. (2019)

System / phase / intent: Enterococcus faecalis ATCC 29212; Human extracted-teeth root-canal biofilm | 30-day mature biofilm followed by 7-day intracanal treatment. Phase E. Viable-cell reduction / endodontic decontamination. Context: Dental / root canal.

Intervention and comparison: Ciprofloxacin; levofloxacin | NAC 8 mg/mL with each antibiotic concentration series. CIP vs CIP+NAC; LEV vs LEV+NAC. Formulation: Free intracanal NAC. NAC-alone: Present.

Exposure: Stock pH — Not reported. Exposure pH — Not reported for the relevant biofilm exposure. pH control — Not identified. Growth medium — BHI. Treatment medium — Intracanal medicaments; final vehicle pH not reported. Hydrodynamics — Batch; agitation not reported.

Outcome / interpretation: CFU/log CFU reduction; SEM biofilm structure. Incremental CFU contrast. Ciprofloxacin and levofloxacin combinations were tested against mature E. faecalis root-canal biofilms; local substrate/exposure cannot be generalized to systemic therapy.

Source: Full text verified; Rastegar Khosravi.pdf; PDF pages 2–3.

## S25 | Bazrgari, F., et al. (2020)

System / phase / intent: Staphylococcus epidermidis; Free and nanoliposomal rifampin/NAC formulations | Both biofilm formation and established-biofilm eradication; 24–72 h exposure. Phase F;E. Formation inhibition + established-biofilm response. Context: S. epidermidis / device-relevant.

Intervention and comparison: Rifampin | Free or liposomal NAC. Rifampin alone vs rifampin+NAC in free and matched liposomal forms. Formulation: Free and liposomal NAC/rifampicin. NAC-alone: Partial / formulation-specific.

Exposure: Stock pH — Not reported. Exposure pH — PBS used for liposome hydration; biofilm exposure pH not reported. pH control — Not identified. Growth medium — BHI preculture; glucose-supplemented MHB biofilm. Treatment medium — Assay broth; liposomal and free-drug arms. Hydrodynamics — Batch; agitation not reported.

Outcome / interpretation: Optical-density ratio; formation inhibition; eradication; time/concentration response. Formulation-matched incremental contrasts. Free and liposomal rifampicin combinations differed by formulation and time; the NAC-specific pharmacologic effect is not identical to the formulation effect.

Verification note: HPLC mobile-phase pH 3 and flow rates are analytical conditions, not biofilm exposure.

Source: Full text verified; Bazrgari.pdf; PDF pages 2–4.

## S26 | Pijls, B. G., et al. (2020)

System / phase / intent: Staphylococcus aureus ATCC 29213; Titanium alloy coupons; induction-heating model | 24-h biofilm followed by 24-h antibiotic/NAC exposure. Phase E. Viable-cell reduction / implant-salvage model. Context: Orthopaedic titanium implant.

Intervention and comparison: Vancomycin + rifampicin regimen | Free NAC. Vancomycin+rifampicin vs same regimen + NAC (no-heat arm). Formulation: Free NAC with vancomycin + rifampicin. NAC-alone: Partial / formulation-specific.

Exposure: Stock pH — Not reported. Exposure pH — Not reported for the relevant biofilm exposure. pH control — Not identified. Growth medium — BHI. Treatment medium — BHI. Hydrodynamics — Batch; agitation not reported.

Outcome / interpretation: CFU/cm²; eradication; heat-antibiotic interaction. No-heat matched multidrug comparison only. The relevant contrast is vancomycin–rifampicin versus the same regimen plus NAC without heating. Heat-associated eradication cannot be attributed to NAC.

Verification note: NAC-alone arm is not available across every heat/no-heat cell. Preserve printed NAC units; verify unusual mg/L dose before converting.

Source: Full text verified; Pijls.pdf; PDF pages 3–5.

## S27 | Ziemyte, M., et al. (2020)

System / phase / intent: S. aureus 240; MRSA4; S. epidermidis 43040; xCELLigence real-time impedance biofilm model | Early established/exponential biofilm: 6 h, 7 h or 9 h depending strain; then 20 h monitoring. Phase E. Early biofilm inhibition / viability. Context: Staphylococcal device-relevant isolates.

Intervention and comparison: Dalbavancin | Free NAC concentration matrix. Dalbavancin alone vs dalbavancin + NAC. Formulation: Free NAC. NAC-alone: Present.

Exposure: Stock pH — Not reported. Exposure pH — Not reported for the relevant biofilm exposure. pH control — Not identified. Growth medium — TSB. Treatment medium — TSB-based impedance/biofilm assays. Hydrodynamics — Agitated batch.

Outcome / interpretation: Cell index/impedance; biofilm mass; viable counts. Impedance/viability contrast; endpoint-specific. NAC could diminish dalbavancin-associated antibiofilm effects in selected staphylococcal conditions.

Verification note: PBS pH 7.4 is a wash, not NAC exposure. Impedance is not directly a viable count.

Source: Full text verified; Ziemyte.pdf; PDF pages 2–4.

## S28 | Öztürk, İ., et al. (2020)

System / phase / intent: Clinical coagulase-negative staphylococci (CoNS) from catheters; Microplate crystal-violet assays; qRT-PCR | Both formation and mature-biofilm assays. Phase F;E. Formation inhibition + established-biofilm response. Context: Clinical coagulase-negative staphylococci.

Intervention and comparison: Gentamicin; ciprofloxacin; doxycycline; rifampicin | Free NAC. Antibiotic alone and NAC+antibiotic conditions. Formulation: Free NAC. NAC-alone: Present.

Exposure: Stock pH — Not reported. Exposure pH — Not reported for the relevant biofilm exposure. pH control — Not identified. Growth medium — TSB + 2.5% glucose. Treatment medium — MHB II / assay-specific broth. Hydrodynamics — Batch; agitation not reported.

Outcome / interpretation: Biofilm inhibition; mature-biofilm response; FICI; icaA/IS256 expression. MIC/FICI and biofilm assays distinguished. NAC combinations differed by antibiotic and isolate; doxycycline and ciprofloxacin were not interchangeable.

Source: Full text verified; O╠ИZtU╠ИRk, .pdf; PDF pages 8–10.

## S29 | Aiyer et al. (2021a)

System / phase / intent: Burkholderia cenocepacia; 1 reference + 5 CF clinical isolates; Static mature biofilm + CLSM | 48-h biofilm followed by 24-h treatment. Phase E. Viable-cell reduction + matrix disruption. Context: Explicit cystic fibrosis.

Intervention and comparison: Ciprofloxacin | Free NAC. Ciprofloxacin alone vs ciprofloxacin + NAC. Formulation: Free intrinsic-pH NAC. NAC-alone: Present.

Exposure: Stock pH — Unneutralized. Exposure pH — NAC-containing TSB/PBS pH measured by concentration. pH control — Acidic citric-acid controls in ancillary assays. Growth medium — TSB. Treatment medium — PBS for mature biofilm treatment. Hydrodynamics — Static.

Outcome / interpretation: CFU/log reduction; CLSM live/dead biovolume. Pairwise CFU comparisons; not a single standardized interaction model. Intrinsic-pH NAC plus ciprofloxacin reduced mature B. cenocepacia burden, but acidification and NAC-alone activity affect attribution.

Verification note: At 30/50 mM NAC, PBS pH was 2.64/2.42; these conditions are not equivalent to neutralized NAC.

Source: Full text verified; Aiyer 2020.pdf; PDF pages 8–11, 13.

## S30 | Aiyer et al. (2021b)

System / phase / intent: B. cenocepacia; Achromobacter xylosoxidans; Stenotrophomonas maltophilia; Static mature-biofilm assays | 48-h biofilms followed by 24-h treatment. Phase E. Viability reduction / mature biofilm treatment. Context: Explicit cystic fibrosis.

Intervention and comparison: Pathogen-appropriate antibiotics including ciprofloxacin, colistin and tobramycin | Free NAC. Corresponding antibiotic alone vs antibiotic + NAC. Formulation: Free neutralized NAC. NAC-alone: Present.

Exposure: Stock pH — TSB stock adjusted to 6.5–7.4. Exposure pH — Not reported for the relevant biofilm exposure. pH control — Not identified. Growth medium — TSB. Treatment medium — TSB / PBS, assay-specific. Hydrodynamics — Static.

Outcome / interpretation: Viable counts; biofilm killing/disruption. Planktonic interaction screening separate from mature-biofilm response. Neutralized NAC combinations varied across B. cenocepacia, A. xylosoxidans and S. maltophilia and across antibiotics.

Verification note: High nominal NAC concentrations are not dose-equivalent to acidic NAC in Aiyer 2021a.

Source: Full text verified; A. Aiyer, S. K. Visser,.pdf; PDF pages 22–24.

## S31 | Birk, S. E., et al. (2021)

System / phase / intent: Pseudomonas aeruginosa; Ciprofloxacin-loaded chitosan microcontainers with CHI/NAC lid | Mucin-embedded biofilm; local-delivery eradication model. Phase E. Localized delivery / viability reduction. Context: Explicit cystic fibrosis model.

Intervention and comparison: Ciprofloxacin | NAC incorporated into functionalized microcontainer lid. Ciprofloxacin microcontainer without NAC vs matched NAC-functionalized system. Formulation: Ciprofloxacin microcontainers with chitosan/NAC lid. NAC-alone: Partial / formulation-specific.

Exposure: Stock pH — Not reported. Exposure pH — Not reported for the relevant biofilm exposure. pH control — Not identified. Growth medium — Diluted artificial sputum medium, mucin-poor/rich. Treatment medium — Artificial sputum medium. Hydrodynamics — Continuous flow.

Outcome / interpretation: Local antibiotic delivery; biofilm bacterial reduction/eradication. Matched delivery platform; not molecular synergy. NAC-containing lids changed local delivery and biofilm response under perfusion; findings are not interchangeable with free-solution assays.

Verification note: 1 µL/min; 48-h mucin-rich or 72-h mucin-poor growth. pH 4 QCM-D mucin experiment is not the biofilm exposure.

Source: Full text verified; Birk.pdf; PDF pages 22–24.

## S32 | Cerutti, J. P., et al. (2021)

System / phase / intent: Staphylococcus aureus (MRSA/MSSA); Free drug and cyclodextrin multicomponent complexes | ~24-h preformed biofilm; post-formation treatment. Phase E. Metabolic inhibition / formulation enhancement. Context: Staphylococcal clinical isolates; no organ-specific model.

Intervention and comparison: Chloramphenicol | Free NAC and NAC-containing γ-cyclodextrin complex. Chloramphenicol alone vs chloramphenicol + NAC; matched γ-CD chloramphenicol complex without NAC. Formulation: Free NAC and cyclodextrin complexes. NAC-alone: Partial / formulation-specific.

Exposure: Stock pH — Complex preparation solutions verified at 6.8. Exposure pH — Not reported for the relevant biofilm exposure. pH control — Not identified. Growth medium — TSB + 0.25% glucose. Treatment medium — Fresh nutrient medium. Hydrodynamics — Agitated batch.

Outcome / interpretation: Biofilm metabolic activity (XTT); viability/antibiofilm effect. Matched binary versus ternary complex. Chloramphenicol–NAC and matched cyclodextrin contrasts evaluate metabolic activity in established staphylococcal biofilms; formulation chemistry contributes to effect.

Verification note: The biofilm protocol reports 335 K. This apparent reporting anomaly is treated as an unresolved source concern.

Source: Full text verified; Juan P. Cerutti.pdf; PDF pages 3–4.

## S33 | Kuyucuklu, G., et al. (2021)

System / phase / intent: 38 S. aureus + 12 S. epidermidis clinical isolates; Microplate biofilm; MBEC/checkerboard | ~18-h formed biofilm before susceptibility testing. Phase E. Biofilm susceptibility / viable-cell reduction. Context: Clinical MRSA.

Intervention and comparison: Ampicillin; vancomycin | Free NAC. Each antibiotic alone vs same antibiotic + NAC. Formulation: Free NAC. NAC-alone: Present.

Exposure: Stock pH — Not reported. Exposure pH — Not reported for the relevant biofilm exposure. pH control — Not identified. Growth medium — TSB / study-specific growth medium. Treatment medium — MHB-based susceptibility exposure. Hydrodynamics — Batch; agitation not reported.

Outcome / interpretation: MBEC; FICI; CFU/log reduction; gene expression. Biofilm MBEC/FICI and CFU outcomes. NAC combinations were evaluated in established MRSA biofilms; interaction classifications apply to the corresponding antibiotic/endpoint rather than the whole paper.

Source: Full text verified; Kuyucuklu.pdf; PDF pages 3–5.

## S34 | Manoharan, A., et al. (2021)

System / phase / intent: Uropathogenic Escherichia coli and Enterococcus faecalis; Static microplate biofilm | 48-h preformed biofilm followed by 24-h treatment. Phase E. Viable-cell reduction; epithelial assays ancillary. Context: Urinary / bladder.

Intervention and comparison: Ciprofloxacin | Free NAC. Ciprofloxacin alone vs ciprofloxacin + NAC. Formulation: Free intrinsic-pH NAC. NAC-alone: Present.

Exposure: Stock pH — Not reported. Exposure pH — Acidic NAC discussed (~2.5); endpoint-specific final pH not consistently given. pH control — Not identified. Growth medium — TSB. Treatment medium — PBS for mature biofilm treatment. Hydrodynamics — Static.

Outcome / interpretation: CFU; membrane damage/PI staining; biofilm killing. Pairwise CFU contrasts; no formal synergy model. Ciprofloxacin plus NAC reduced mature uropathogen burden; substantial NAC-alone activity and an insignificant increment in some comparisons constrain synergy claims.

Verification note: NAC-only epithelial/formation assays are not counted as antibiotic-combination formation outcomes.

Source: Full text verified; Arthika Manoharan 1, Samantha Ognenovska.pdf; PDF pages 12–14.

## S35 | Pinto, R. M., et al. (2021)

System / phase / intent: P. aeruginosa ATCC 27853; S. epidermidis ATCC 35984; Free drug + NAC-loaded/unloaded lipid nanoparticles functionalized with D-amino acids | Mature P. aeruginosa (2 d) and S. epidermidis (3 d) biofilms; post-formation treatment. Phase E. Matrix/biomass disruption + metabolic viability. Context: Staphylococcal and Pseudomonas biofilms; no site-specific host model.

Intervention and comparison: Moxifloxacin | Free NAC or NAC-loaded functionalized LNPs. Free MOX vs MOX+NAC; unloaded vs NAC-loaded matched LNP adjuncts. Formulation: Free NAC and NAC-loaded lipid nanoparticle adjuncts. NAC-alone: Partial / formulation-specific.

Exposure: Stock pH — NAC dissolved using NaOH during particle preparation. Exposure pH — Not reported for the relevant biofilm exposure. pH control — Not identified. Growth medium — TSB. Treatment medium — Assay-specific broth. Hydrodynamics — Agitated batch.

Outcome / interpretation: Biofilm biomass; bacterial viability; formulation safety. Matched loaded/unloaded adjunct contrasts. Carrier effects and NAC loading altered biofilm biomass and moxifloxacin responses; unloaded vehicles themselves were active.

Verification note: Preparation with NaOH is not a measured neutral biofilm exposure. Biomass and metabolic endpoints can diverge.

Source: Full text verified; Pinto 2021.pdf; PDF pages 3–5.

## S36 | Son et al. (2021)

System / phase / intent: 14 clinical Pseudomonas aeruginosa isolates from canine otitis externa; 96-well crystal-violet biofilm formation assay | Formation/inhibition model; 12 h. Phase F. Formation prevention / biomass inhibition. Context: Veterinary / canine otitis externa.

Intervention and comparison: Enrofloxacin; polymyxin B; gentamicin | Free NAC. Each antibiotic alone vs same antibiotic + NAC. Formulation: Free NAC. NAC-alone: Present.

Exposure: Stock pH — Not reported. Exposure pH — Not reported for the relevant biofilm exposure. pH control — Not identified. Growth medium — TSB. Treatment medium — TSB. Hydrodynamics — Agitated batch.

Outcome / interpretation: MIC/FICI; biofilm biomass (OD570). Planktonic FICI; biofilm biomass separately assessed. NAC inhibited formation, but the report did not establish significant synergy with enrofloxacin, gentamicin or polymyxin B.

Verification note: Do not include this as mature-biofilm polymyxin potentiation.

Source: Full text verified; Son.pdf; PDF pages 2–5.

## S37 | Alarfaj, R. E., et al. (2022)

System / phase / intent: Tobramycin-resistant E. coli, K. pneumoniae and A. baumannii; six representative isolates in biofilm figures; Preformed biofilms in 96-well plates; 75-rpm growth; 24-h treatment. Section 2.7 mentions 72 h, a further 48 h, then 72 h again; exact pretreatment age is internally inconsistent. Phase E. Established-biofilm biomass reduction and liposomal delivery enhancement; separate planktonic inhibition/killing endpoints, not demonstrated biofilm killing. Context: Drug-resistant Gram-negative clinical isolates; liposomal delivery, not a clinical treatment study.

Intervention and comparison: Tobramycin liposomes (TL) versus tobramycin-NAC liposomes (TNL); NAC 50 mM used in preparation and as free NAC control. Biofilm doses use formulation-specific MICs, not universally equal tobramycin doses.. TL versus TNL with same reported tobramycin dose only for EC_219 (32 mg/L) and KP_059 (16 mg/L) in Figures 5 and 7. Four other figure comparisons use unequal MIC-linked doses and are contextual, not dose-matched NAC contrasts.. Formulation: TL/TNL liposomes: DMPE/DPPC/cholesterol 4:2:1; 1 mg tobramycin with/without 50 mM NAC during preparation; particle size and encapsulation differ; delivered/released NAC exposure not quantified. NAC-alone: Partial/formulation-specific: free NAC 50 mM control present; a NAC-only liposome control not reported.

Exposure: Stock pH — Preparation uses PBS stated as pH 7.4; NAC-containing preparation pH after mixing not separately measured/reported. Exposure pH — Final Mueller-Hinton biofilm-treatment pH not reported; preparation PBS pH is not final exposure pH. pH control — No acid-only or pH-matched biofilm-treatment control reported. Growth medium — Mueller-Hinton broth; 96-well preformed-biofilm model. Treatment medium — Mueller-Hinton-based treatment assay; formulation diluted at its MIC; final treatment chemistry/pH not measured. Hydrodynamics — Growth in shaking incubator at 75 rpm; 24-h treatment agitation not specified. Batch culture, not continuous flow..

Outcome / interpretation: Crystal-violet residual biomass, OD570, after established-biofilm exposure; planktonic MIC/MBC reported separately; formulation size/encapsulation/release. Formulation/biomass comparison without a formal biofilm interaction model. MIC reductions are planktonic; most biofilm TL/TNL comparisons are not dose matched.. TNL reduced biomass relative to untreated control, but it did not uniformly improve on TL. In dose-matched Figure 5 (EC_219, 32 mg/L), the TNL mean bar is higher than TL; in Figure 7 (KP_059, 16 mg/L), it is lower. No TL-versus-TNL pairwise significance is shown in those figure annotations. Four other isolate comparisons use unequal tobramycin doses. Retain as formulation-specific, conditional evidence, not uniform NAC potentiation or biofilm synergy.

Verification note: Six biofilm isolates are distinct from the 21-isolate susceptibility panel. Preparation pH 7.4 is not final exposure pH; 50 mM preparation NAC is not a measured intrabiofilm NAC dose. Biofilm-age chronology is inconsistent. Figures 4-9 compare untreated control with TNL, not an explicit TL/TNL interaction test. Visual directions were checked without graph digitization.

Source: Full text verified; Alarafaj 2022(1).pdf; PDF pages 3 (preparation); 4-5 (biofilm protocol/replication); 6-7 (size, encapsulation, MICs); 9-12 (Figures 4-9).

## S38 | Kuruoglu, T., et al. (2022)

System / phase / intent: Methicillin-resistant Staphylococcus aureus (MRSA); Ventriculoperitoneal shunt segments | 24-h biofilm followed by 24-h treatment. Phase E. Established shunt-biofilm viable-cell reduction. Context: Neurosurgical shunt.

Intervention and comparison: Daptomycin; vancomycin; linezolid | Free NAC added to each antibiotic regimen. Each antibiotic alone vs same antibiotic + NAC. Formulation: Free NAC; impregnated and nonimpregnated shunts. NAC-alone: Present.

Exposure: Stock pH — Not reported. Exposure pH — Not reported for the relevant biofilm exposure. pH control — Not identified. Growth medium — Plasma conditioning; TSB + 0.5% glucose. Treatment medium — MHB; calcium supplemented for daptomycin. Hydrodynamics — Batch; agitation not reported.

Outcome / interpretation: Biofilm-associated bacterial counts / eradication. Within-shunt-type matched contrast. Responses to daptomycin, vancomycin and linezolid with NAC depended on the pre-impregnated versus nonimpregnated substrate.

Verification note: Rifampicin/clindamycin in some shunts is a background co-intervention, not a standalone rifampicin–NAC experiment.

Source: Full text verified; Kuruoglu 2022.pdf; PDF pages 2–4.

## S39 | Llamosi, M., et al. (2022)

System / phase / intent: Streptococcus pneumoniae; Microplate biofilm assays | Early established biofilm (~4 h) plus prevention/disaggregation assays. Phase F;E. Formation prevention + disaggregation/viable-cell reduction. Context: Respiratory / pneumococcal.

Intervention and comparison: Cefditoren | Free NAC. Cefditoren alone vs cefditoren + NAC. Formulation: Free NAC. NAC-alone: Present.

Exposure: Stock pH — Not reported. Exposure pH — C+Y medium based on CpH8; final pH after NAC not measured. pH control — Not identified. Growth medium — C+Y (CpH8 + 0.08% yeast extract). Treatment medium — C+Y; RPMI/HEPES only for epithelial adhesion assay. Hydrodynamics — Batch; agitation not reported.

Outcome / interpretation: Biofilm viability; disaggregation; CFU/antibiofilm activity. Biofilm CFU/disaggregation comparisons; assay-specific. Cefditoren–NAC affected early pneumococcal biofilm development and preformed biofilms; host adhesion results are a separate endpoint.

Source: Full text verified; Llamosi.pdf; PDF pages 11–12.

## S40 | Mansouri, M. D., et al. (2022)

System / phase / intent: MRSE; levofloxacin-sensitive MRSA; levofloxacin-resistant MRSA; VRE; E. coli; K. pneumoniae; P. aeruginosa (no MSSA arm reported); Catheter lumens colonized for 24 h, followed by 12-h lock treatment; separate 24-96-h durability assay and SEM of colonized catheter segments Phase E. Established catheter-associated viable-burden reduction and structural disruption for experimental salvage; separate durability and catheter-material compatibility endpoints. Context: Vascular catheter-associated infection / experimental catheter salvage; not CF.

Intervention and comparison: Levofloxacin 5 mg/mL with/without NAC 10 mg/mL, prepared in normal saline; fixed concentrations for the main four-arm comparison. 12-h assay: levofloxacin 5 mg/mL alone versus same levofloxacin + NAC 10 mg/mL; NAC-only and saline controls. Durability assay compares combination with saline only, not with antibiotic alone.. Formulation: Free NAC 10 mg/mL (Sigma-Aldrich) + levofloxacin 5 mg/mL (Sigma-Aldrich) in normal-saline catheter lock. NAC-alone: Present in main 12-h quantitative and SEM experiments; absent, together with levofloxacin-only arm, from extended durability comparison.

Exposure: Stock pH — No NAC stock pH, neutralization or added buffer reported. Exposure pH — Final saline lock-solution pH not reported. pH control — No acid-only or pH-matched lock control reported. Growth medium — Trypticase soy broth (TSB); overnight cultures and 24-h catheter colonization. Treatment medium — Normal saline; nutrient-poor lock exposure; control decline attributed by authors partly to nutrient absence. Hydrodynamics — Locked-lumen batch dwell without perfusion in main assay; separate SEM specimens colonized on a rocker. Mixed stationary/rocker batch components, not continuous flow..

Outcome / interpretation: Recovered adherent CFU/cm after sonication/vortexing; SEM structure; 24-96-h culture recovery; catheter force-at-break after 30 days (material, not patient safety). Main four-arm matched-concentration viable-count comparison; no formal interaction model. Extended durability is combination-versus-saline only.. At 12 h, Figure 1 supports lower combination CFU than levofloxacin alone for MRSE, both MRSA phenotypes, P. aeruginosa and E. coli; VRE and K. pneumoniae levofloxacin-versus-combination differences are marked NS. The 24-96-h assay reports no recovered P. aeruginosa/E. coli at 24 h and MRSA at 96 h, but lacks monotherapy comparators; MRSE remains recoverable. These observations do not prove uniform synergy, a monotherapy-relative durability benefit, or clinical catheter salvage.

Verification note: The source tested levofloxacin-sensitive and levofloxacin-resistant MRSA, not MSSA. Main and durability assays used different control sets. NS in Figure 1 does not establish equivalence. No-colony recovery is assay-specific; a quantitative detection limit and post-withdrawal regrowth test were not reported. Catheter force-at-break testing concerns material compatibility rather than clinical safety. Nested segments do not represent 20 independent isolates.

Source: Full text verified; Mansouri 2022(1).pdf; PDF pages 2-3 (methods, controls, doses, medium, replication); 4 (Figure 1); 4-5 (durability, Figure 2); 6-8 (SEM, material testing, discussion).

## S41 | Pinto, R. M., et al. (2022)

System / phase / intent: Staphylococcus aureus (including MRSA/MSSA strains); MOX-loaded multiple lipid nanoparticles (MLNs) + NAC-loaded LNPs; microtiter and polyurethane catheter | Mature biofilms. Phase E. Biomass/viability reduction; catheter delivery. Context: Catheter / staphylococcal biofilms.

Intervention and comparison: Moxifloxacin | Separate NAC-loaded LNP adjunct. MOX-loaded MLN without NAC adjunct vs MOX-loaded MLN + NAC-loaded LNP; unloaded-LNP controls. Formulation: NAC-loaded lipid nanoparticle adjunct to moxifloxacin particles. NAC-alone: Partial / formulation-specific.

Exposure: Stock pH — Particle preparation as described; no final assay pH. Exposure pH — Not reported for the relevant biofilm exposure. pH control — Not identified. Growth medium — 0.6× TSB + 0.2% glucose. Treatment medium — Assay-specific nutrient broth. Hydrodynamics — Batch; agitation not reported.

Outcome / interpretation: Biofilm biomass; viability; catheter biofilm activity; microscopy. Matched loaded/unloaded carrier adjuncts. Moxifloxacin-particle responses depended on the adjunct carrier; NAC-alone and unloaded-particle effects must be separated.

Verification note: Shaking in overnight preculture, staining or XTT development is not proof that treatment was agitated.

Source: Full text verified; Pinto.pdf; PDF pages 3–6.

## S42 | Valzano, F., et al. (2022)

System / phase / intent: Pseudomonas aeruginosa; 17 strains (15 from CF patients); Nunc-TSP peg biofilm; additional artificial sputum medium (ASM) model | Preformed biofilms; post-formation challenge. Phase E. Viable-cell reduction / established-biofilm treatment. Context: Explicit cystic fibrosis.

Intervention and comparison: Colistin | Free NAC, including high inhalation-achievable concentrations. Colistin alone vs colistin + NAC. Formulation: Free neutralized NAC. NAC-alone: Present.

Exposure: Stock pH — Adjusted to 6.5–6.8 with NaOH. Exposure pH — Not reported for the relevant biofilm exposure. pH control — Not identified. Growth medium — CAMHB pegs; artificial sputum medium aggregates. Treatment medium — CAMHB or artificial sputum medium. Hydrodynamics — Static.

Outcome / interpretation: CFU/peg; eradication; synergy; ASM confirmation; NAC transcriptomics (secondary). Separate viable-count contrasts in two media. Colistin–NAC activity differed between peg and sputum-medium models; selected ASM experiments required higher colistin exposure than peg assays.

Verification note: NAC-alone effects were strain-dependent. Transcriptomics and time-kill studies were planktonic and are not biofilm-mechanism proof.

Source: Full text verified; Valzano.pdf; PDF pages 15–17.

## S43 | Huang, Z., et al. (2023)

System / phase / intent: Ceftazidime-avibactam-resistant Enterobacterales; Microplate biofilm assays | Biofilm formation and already-formed/mature biofilm assays. Phase F;E. Formation inhibition + biomass disruption. Context: Resistant K. pneumoniae; infection model ancillary.

Intervention and comparison: Ceftazidime-avibactam (CZA) | Free NAC. CZA alone vs CZA + NAC. Formulation: Free NAC with ceftazidime–avibactam. NAC-alone: Present.

Exposure: Stock pH — Not reported. Exposure pH — Not reported for the relevant biofilm exposure. pH control — Not identified. Growth medium — LB. Treatment medium — LB. Hydrodynamics — Batch; agitation not reported.

Outcome / interpretation: Antibacterial susceptibility; biofilm formation; mature-biofilm activity. Planktonic FICI/time-kill; biofilm biomass separate. Ceftazidime–avibactam plus NAC reduced biofilm biomass in formation and preformed assays; mouse outcomes do not replace biofilm-specific evidence.

Source: Full text verified; Huang 2023.pdf; PDF pages 8–9.

## S44 | Lima, E. M. F., et al. (2023)

System / phase / intent: Pseudomonas aeruginosa PAO1; 96-well microplate and coverslip biofilm formation models | Formation/inhibition model; 24 h. Phase F. Formation prevention / quorum-sensing phenotype. Context: P. aeruginosa laboratory model.

Intervention and comparison: Tobramycin | Free NAC (concentration matrix). Tobramycin alone vs tobramycin + NAC. Formulation: Free NAC. NAC-alone: Present.

Exposure: Stock pH — Not reported. Exposure pH — Not reported for the relevant biofilm exposure. pH control — Not identified. Growth medium — LB. Treatment medium — LB. Hydrodynamics — Batch; agitation not reported.

Outcome / interpretation: Crystal-violet biofilm formation; SynergyFinder interaction analysis; CLSM; quorum-sensing phenotypes. SynergyFinder applied to formation biomass; planktonic FICI separate. NAC–tobramycin showed an interaction in a biofilm-formation assay; this is not established-biofilm eradication.

Verification note: Acidification to pH 2 is a rhamnolipid extraction step, not NAC treatment pH.

Source: Full text verified; Lima.pdf; PDF pages 2–4.

## S45 | Petkova, T., et al. (2023)

System / phase / intent: S. aureus ATCC 25923; S. aureus O74; E. coli ATCC 25922; P. aeruginosa ATCC 27853; Safranin microplate biofilm assay | Biofilm formation / MBIC; 48-h cultivation. Phase F. Formation prevention / biomass inhibition. Context: Veterinary-oriented; multiple reference pathogens.

Intervention and comparison: Doxycycline | Free NAC. Doxycycline alone vs doxycycline + NAC. Formulation: Free NAC. NAC-alone: Present.

Exposure: Stock pH — Prepared in TSB/MHB without reported adjustment. Exposure pH — Not reported for the relevant biofilm exposure. pH control — Not identified. Growth medium — TSB. Treatment medium — TSB. Hydrodynamics — Agitated then static.

Outcome / interpretation: MIC; MBIC; biofilm biomass. MBIC changes; no formal biofilm interaction model. Doxycycline–NAC results differed among species; low NAC exposure did not show consistent benefit and planktonic growth stimulation was reported in S. aureus.

Verification note: 24 h at 150 rpm then 24 h without shaking. HCl/NaOH belong to safranin processing, not NAC buffering.

Source: Full text verified; Petkova.pdf; PDF pages 8–9.

## S46 | Alhajj, N., et al. (2024)

System / phase / intent: Pseudomonas aeruginosa ATCC 10145; Spray-dried inhalable co-amorphous powder | 48-h biofilm; treatment/inhibition assessment. Phase F;E. Formation inhibition + established-biofilm biomass response. Context: Pulmonary delivery / CF-motivated formulation.

Intervention and comparison: Ciprofloxacin + quercetin regimen | NAC incorporated into ternary spray-dried CIP–quercetin–NAC powder (SD-CQN). Matched CIP + quercetin powder (SD-CQ) without NAC. Formulation: Spray-dried ciprofloxacin–quercetin ± NAC. NAC-alone: Not identified.

Exposure: Stock pH — Not reported. Exposure pH — Not reported for the relevant biofilm exposure. pH control — Not identified. Growth medium — LB. Treatment medium — LB with matched DMSO exposure. Hydrodynamics — Batch; agitation not reported.

Outcome / interpretation: Antibacterial activity; biofilm inhibition; physicochemical and aerosolization properties. Matched multicomponent formulation contrast. Adding NAC altered a ciprofloxacin–quercetin formulation and its biofilm activity; quercetin, solid state and delivery preclude a free NAC–ciprofloxacin synergy inference.

Verification note: PBS pH 7.4 belongs to dissolution testing; it does not establish final LB exposure pH.

Source: Full text verified; Alhajj.pdf; PDF pages 4–5.

## S47 | Higazy, D., et al. (2024)

System / phase / intent: Pseudomonas aeruginosa; Flow-cell biofilm | 72-h biofilm before ciprofloxacin ± antioxidant exposure. Phase R. Resistance-evolution modulation. Context: Explicit CF-motivated P. aeruginosa research.

Intervention and comparison: Ciprofloxacin | Free NAC antioxidant co-treatment. Ciprofloxacin alone vs ciprofloxacin + NAC. Formulation: Free NAC. NAC-alone: Not identified for every evolution contrast.

Exposure: Stock pH — Not reported. Exposure pH — Not reported for the relevant biofilm exposure. pH control — Not identified. Growth medium — Flow-cell medium; LB in glass-bead model. Treatment medium — Ciprofloxacin/antioxidant-containing growth medium. Hydrodynamics — Continuous flow + agitated batch.

Outcome / interpretation: Resistance evolution; population susceptibility; resistance-associated mutations. Repeated-passage resistance endpoints, not acute synergy. NAC-associated resistance effects depended on flow-cell versus bead models and were weaker than those of some other antioxidants.

Verification note: Do not label all model-specific effects as suppression of resistance or acute antibiofilm benefit.

Source: Full text verified; Higazy The impact.pdf; PDF pages 8–10.

## S48 | Karpova, E. V., et al. (2024)

System / phase / intent: Streptococcus pneumoniae; Staphylococcus aureus; Klebsiella pneumoniae; 96-well microtiter biofilm formation; crystal violet | Formation/inhibition model; 24-h cultivation. Phase F. Formation prevention / biomass response. Context: Respiratory pathogens; thiamphenicol formulation.

Intervention and comparison: Thiamphenicol; chloramphenicol; thiamphenicol glycinate acetylcysteinate | Free NAC with thiamphenicol/chloramphenicol; acetylcysteinate-containing derivative. Antibiotic alone vs matched antibiotic + NAC / acetylcysteinate derivative. Formulation: Free NAC and thiamphenicol glycinate acetylcysteinate. NAC-alone: Present.

Exposure: Stock pH — Not reported. Exposure pH — Not reported for the relevant biofilm exposure. pH control — Not identified. Growth medium — MHB and brain–heart broth. Treatment medium — MHB and brain–heart broth. Hydrodynamics — Batch; agitation not reported.

Outcome / interpretation: MIC; biofilm optical density/biomass; viable-cell counts. Free-mixture and chemical-form comparisons distinguished. NAC combinations altered biofilm formation in a species-dependent fashion, including countering antibiotic-associated biomass increases.

Verification note: A chemically defined acetylcysteinate preparation is not automatically equivalent to adding free NAC.

Source: Full text verified; Karpova.pdf; PDF pages 2–5.

## S49 | Aguilar-Colomer, A., et al. (2025)

System / phase / intent: Escherichia coli; Mesoporous silica nanocarrier with gelatin outer layer | 48-h mature biofilm; post-formation treatment. Phase E. Viability reduction + matrix disruption / delivery. Context: Local nanocarrier; E. coli model.

Intervention and comparison: Levofloxacin | NAC embedded in gelatin layer; sequential release before levofloxacin. MSN-L@GelL-AC vs matched levofloxacin nanocarrier MSN-L@GelL. Formulation: NAC-containing levofloxacin silica/gelatin system. NAC-alone: Partial / formulation-specific.

Exposure: Stock pH — Not reported. Exposure pH — Not reported for the relevant biofilm exposure. pH control — Not identified. Growth medium — LB; sucrose supplementation. Treatment medium — Nanoparticle exposure solution; see source. Hydrodynamics — Agitated batch.

Outcome / interpretation: Biofilm viability/eradication; antimicrobial efficacy; microscopy/biocompatibility. Matched platform comparisons; release confounding retained. Levofloxacin nanocarrier comparisons favored selected NAC-containing systems; altered release and gelatin effects remain part of the formulation contrast.

Verification note: Biofilm growth/treatment 100 rpm. pH 7.4 reported for PBS release/stability is not post-addition exposure pH.

Source: Full text verified; Aguilar-Colomer.pdf; PDF pages 3–4.

## S50 | Altunova et al. (2025)

System / phase / intent: Clinical staphylococci; S. aureus reference strains included; Microplate formed-biofilm susceptibility testing; MBIC/checkerboard | Established/formed biofilm. Phase E. Suppression of biofilm-derived regrowth. Context: Clinical MRSA / coagulase-negative staphylococci.

Intervention and comparison: Vancomycin | Free NAC among tested mucolytics. Vancomycin alone vs vancomycin + NAC. Formulation: Free NAC. NAC-alone: Present.

Exposure: Stock pH — Dissolved in water; no adjustment reported. Exposure pH — Not reported for the relevant biofilm exposure. pH control — Not identified. Growth medium — TSB + 1% glucose. Treatment medium — CAMHB. Hydrodynamics — Batch; agitation not reported.

Outcome / interpretation: MIC; MBIC; checkerboard/FICI; biofilm-associated gene analysis. FICI based on biofilm MBIC (not planktonic MIC). Vancomycin–NAC interactions were calculated from a preformed-biofilm regrowth endpoint and varied across staphylococcal isolates.

Verification note: MBIC in this study is growth after treating preformed biofilm; retain definition rather than equating with formation prevention.

Source: Full text verified; Altunova.pdf; PDF pages 3–4.

## S51 | Josef, M., et al. (2025)

System / phase / intent: Pseudomonas aeruginosa clinical isolate; Ceftazidime-loaded invasomes and nanostructured lipid carriers (NLCs); polystyrene plate | Biofilm formation assay plus 24-h mature biofilm detachment assay followed by 24-h treatment. Phase F;E. Formation prevention + biomass detachment. Context: Ocular / bacterial keratitis.

Intervention and comparison: Ceftazidime | Free NAC combined with CTZ-loaded nanocarriers / aqueous CTZ. CTZ-loaded carrier alone vs same CTZ-loaded carrier + NAC; free CTZ comparator. Formulation: Ceftazidime-loaded lipid/invasome systems ± NAC. NAC-alone: Partial / formulation-specific.

Exposure: Stock pH — Not reported. Exposure pH — Not reported for the relevant biofilm exposure. pH control — Not identified. Growth medium — MHB-based biofilm protocol. Treatment medium — Formulation-containing assay medium. Hydrodynamics — Static.

Outcome / interpretation: Biofilm inhibition; mature-biofilm detachment; crystal violet. Matched carrier contrasts; no formal biofilm interaction rule identified. Ceftazidime formulations plus NAC affected formation and residual biomass of established biofilms; detachment is not verified sterilization.

Verification note: PBS pH 7.2 washing and pH 7.4 release medium do not establish biofilm exposure pH.

Source: Full text verified; Josef.pdf; PDF pages 9–10.

## S52 | Polo-Montalvo, A., et al. (2025)

System / phase / intent: Staphylococcus aureus; Levofloxacin-loaded mesoporous bioactive glass nanoparticles (MBGN) | Mature biofilm; post-formation treatment. Phase E. Viable-cell reduction / biomaterial delivery. Context: Bone-regeneration / orthopaedic local delivery.

Intervention and comparison: Levofloxacin | NAC covalently functionalized on MBGN surface. MBGN-L-NAC vs MBGN-L; released levofloxacin standardized for comparison. Formulation: NAC-functionalized levofloxacin bioactive glass. NAC-alone: Not identified as an NAC-only matched biofilm arm.

Exposure: Stock pH — Not reported. Exposure pH — Not reported for the relevant biofilm exposure. pH control — Not identified. Growth medium — Todd–Hewitt broth + 1% sucrose. Treatment medium — Nanoparticle exposure; release calibration in PBS. Hydrodynamics — Agitated batch.

Outcome / interpretation: Biofilm viability/integrity; antibacterial activity; microscopy; drug release. Released-levofloxacin-normalized formulation comparison. NAC-functionalized and nonfunctionalized particles differed in release and biofilm activity; antibiotic normalization improves but does not eliminate material confounding.

Verification note: PBS pH 6.0/7.4 and SBF pH 7.4→8.1 are release/bioactivity measurements, not final biofilm-treatment pH.

Source: Full text verified; Polo-Montalvo.pdf; PDF pages 5–6, 12.

## S53 | Higazy, D., et al. (2026)

System / phase / intent: Pseudomonas aeruginosa PAO1; Synthetic cystic-fibrosis sputum medium (SCFM2); aggregated biofilm model | 24-h aggregates established before exposure; serial evolution over 6 passages. Phase R. Resistance-evolution modulation. Context: Explicit cystic fibrosis sputum model.

Intervention and comparison: Ciprofloxacin | Free NAC as antioxidant co-treatment. Ciprofloxacin alone vs ciprofloxacin + NAC. Formulation: Free NAC. NAC-alone: Not identified for the evolution contrast.

Exposure: Stock pH — Not reported. Exposure pH — Not reported for the relevant biofilm exposure. pH control — Not identified. Growth medium — SCFM2 after LB preculture. Treatment medium — SCFM2. Hydrodynamics — Static.

Outcome / interpretation: Population analysis profiles; MIC; whole-genome sequencing; resistance-associated mutations. Six-passage population MIC/PAP/WGS; not acute synergy. Antioxidant effects on resistance evolution differed in SCFM2; NAC cannot be described as uniformly preventing ciprofloxacin resistance.

Verification note: 24-h aggregates precede treatment. NAC 2 mM; do not transfer flow-cell conclusions to SCFM2.

Source: Full text verified; Higazy.pdf; PDF pages 3–5, 10–11.

## S54 | Wang, F., et al. (2026)

System / phase / intent: Klebsiella pneumoniae; 7 reference strains + 27 clinical isolates; 96-well microtiter susceptibility/biofilm-formation assay; crystal violet | Formation/inhibition model; ~20 h. Phase F. Formation prevention / biomass inhibition. Context: K. pneumoniae clinical/reference strains.

Intervention and comparison: Polymyxin B | Free acidic NAC; neutralized-NAC arms used mechanistically. Polymyxin B alone vs polymyxin B + NAC. Formulation: Free intrinsic-pH NAC; neutralized comparator in susceptibility assays. NAC-alone: Present.

Exposure: Stock pH — Neutralized comparison 6.8–7.2 with NaOH. Exposure pH — Intrinsic acidity assessed; full per-well exposure-pH series not given. pH control — Neutralized-NAC comparator; not acid-only control for every combination. Growth medium — Broth microdilution medium not explicitly named. Treatment medium — Broth susceptibility/biofilm plate medium. Hydrodynamics — Batch; agitation not reported.

Outcome / interpretation: MIC; FICI; biofilm biomass/inhibition by crystal violet. Planktonic FICI; formation biomass separate. Polymyxin B–NAC was additive in selected nonsusceptible strains and indifferent in susceptible strains; biomass inhibition is not mature-biofilm killing.

Verification note: 34 strains screened; detailed combination classifications apply to selected backgrounds, not a pooled universal effect.

Source: Full text verified; Wang Fei .pdf; PDF pages 2–5.

## S55 | Aksoy et al. (2022)

System / phase / intent: 8 catheter-derived isolates: 3 E. coli, 3 P. mirabilis, 1 P. aeruginosa, 1 P. putida; 96-well plate; 24-h preformed biofilm, then 24-h exposure. Phase E. Residual biomass reduction / MBIC. Context: Paediatric catheter-derived isolates; microplate assay.

Intervention and comparison: Amikacin, cefepime, ceftazidime, colistin, meropenem, piperacillin–tazobactam; NAC 75 mg/mL. Each antibiotic at the same concentration without NAC. Formulation: Free NAC. NAC-alone: Not identified.

Exposure: Stock pH — Fresh aqueous solution with heating; no adjustment reported. Exposure pH — Not reported for the relevant biofilm exposure. pH control — Not identified. Growth medium — TSB + 1% glucose. Treatment medium — CAMHB. Hydrodynamics — Batch; agitation not reported.

Outcome / interpretation: Crystal-violet residual biomass; author-defined MBIC. MBIC shift; no formal interaction model. NAC reduced biofilm inhibitory concentrations for several antibiotic–isolate pairs, with unchanged exceptions. Crystal-violet biomass does not establish bactericidal synergy.

Verification note: 75 mg/mL NAC. Table/text disagreements in some MIC/MBIC entries are not propagated quantitatively; no clinical breakpoint reclassification.

Source: Full text verified; Aksoy.pdf; PDF pages 2–4.

# S5. Focused comparison-level synthesis

Twenty-five focused records below address the most consequential distinctions raised by review. They are purposive explanatory comparisons, not a selected set used to estimate an efficacy rate. The complete study map, including null/unfavorable observations, remains in S4.

## C01 | P. aeruginosa; ciprofloxacin ± NAC; E

Conditions: Stock pH 7.2; final exposure pH not reported; TSB; batch

Endpoint and controls: Metabolic viability estimate + architecture; NAC-alone present

Added activity at selected concentrations is not proof that all effects persist at neutral final pH; MTT-derived burden is not direct plating of every treated biofilm.

Source: Zhao et al. (2010); Zhao.pdf; PDF pages 5–7

## C02 | A. baumannii; colistin ± NAC; E

Conditions: Stock neutralized to 6.5–6.8; CAMHB; static treatment

Endpoint and controls: Viable-burden / biofilm interaction experiments; NAC-alone present

Selected strains and dose combinations support added activity; distinguish resistance phenotype and retain the tested concentrations.

Source: Pollini, S., et al. (2018); Pollini 2018.pdf; PDF pages 2–3, 6

## C03 | S. maltophilia; colistin ± NAC; E

Conditions: Stock neutralized to 6.5–6.8; CAMHB; static peg assay

Endpoint and controls: Viable-burden endpoints; NAC-alone present

Organism-specific evidence cannot establish a uniform polymyxin-class response.

Source: Ciacci, N., et al. (2019); Ciacci.pdf; PDF pages 10–11

## C04 | P. aeruginosa; colistin ± NAC; E; peg model

Conditions: Stock neutralized to 6.5–6.8; CAMHB

Endpoint and controls: Peg biofilm viability; NAC-alone present

Use alongside, not interchangeably with, the artificial-sputum comparison in C05.

Source: Valzano, F., et al. (2022); Valzano.pdf; PDF pages 15–17

## C05 | P. aeruginosa; colistin ± NAC; E; sputum model

Conditions: Same reported NAC stock preparation; artificial sputum medium; selected strains

Endpoint and controls: Viability; NAC-alone present

Selected sputum experiments required colistin 64 mg/L with NAC 8 g/L for the reported strong combination response; lower colistin exposures did not reproduce it. No clinical dose-sparing inference.

Source: Valzano, F., et al. (2022); Valzano.pdf; PDF pages 15–17

## C06 | K. pneumoniae; polymyxin B ± NAC; F

Conditions: Intrinsic-acidic versus neutralized preparations; reported neutral range 6.8–7.2

Endpoint and controls: Crystal-violet formation endpoint; FICI primarily planktonic; NAC-alone present

Acidity and resistance background alter response. Additive planktonic results in selected nonsusceptible groups are not mature-biofilm synergy.

Source: Wang, F., et al. (2026); Wang Fei .pdf; PDF pages 2–5

## C07 | Canine P. aeruginosa; several antibiotics including polymyxin B ± NAC; F

Conditions: pH not reported; broth batch model

Endpoint and controls: Formation endpoint; NAC-alone present

The report does not support significant synergy; this prevention experiment cannot strengthen a claim of mature-biofilm polymyxin potentiation.

Source: Son et al. (2021); Son.pdf; PDF pages 2–5

## C08 | B. cenocepacia; ciprofloxacin ± NAC; E

Conditions: Intrinsic-acidic NAC; pH measured in PBS/TSB; mature exposure in PBS

Endpoint and controls: Killing and structural endpoints; NAC-alone present

Direct NAC activity and acidic/starvation exposure remain part of the intervention; do not pool with a neutralized-stock study.

Source: Aiyer et al. (2021a); Aiyer 2020.pdf; PDF pages 8–11, 13

## C09 | Emerging CF pathogens; ciprofloxacin/colistin/tobramycin ± NAC; E

Conditions: NAC stock adjusted to pH 6.5–7.4; TSB; final per-well pH not established

Endpoint and controls: Biofilm response across organism/drug combinations; NAC-alone present

Results vary across organisms and drugs; stock adjustment is not a pH-matched mechanistic control.

Source: Aiyer et al. (2021b); A. Aiyer, S. K. Visser,.pdf; PDF pages 22–24

## C10 | P. intermedia; ampicillin/ciprofloxacin/tetracycline ± NAC; E

Conditions: Unadjusted NAC; growth-pH and acidity experiments in selected assays

Endpoint and controls: Biofilm/growth response; NAC-alone present

Reduced antibiotic activity is relevant to these drug-specific comparisons, not every antibiotic tested in the paper.

Source: Moon, J. H., et al. (2015); Moon.pdf; PDF pages 2–4, 7

## C11 | P. intermedia; metronidazole ± NAC; E

Conditions: Same source; unadjusted NAC

Endpoint and controls: Biofilm/growth response; NAC-alone present

Metronidazole was not assigned the antagonistic label belonging to other drugs.

Source: Moon, J. H., et al. (2015); Moon.pdf; PDF pages 2–4, 7

## C12 | S. epidermidis; rifampicin ± NAC; E

Conditions: TSB; agitated 130 rpm; final exposure pH not reported

Endpoint and controls: Viability/biomass; NAC-alone present

Mechanistic comparison with rifampicin alone; does not test addition to an active clinical multidrug regimen.

Source: Leite et al. (2013c); B. Leite, F. Gomes.pdf; PDF pages 2–4

## C13 | Staphylococci; rifampicin + clindamycin ± NAC; E

Conditions: NAC neutralized with NaOH; exact target not stated; batch medium per source

Endpoint and controls: Established-biofilm killing; NAC-alone present

The background is a two-antibiotic regimen; assess the NAC increment within that background.

Source: Belfield, K., et al. (2017); Belfield.pdf; PDF pages 2–4

## C14 | S. aureus; vancomycin + rifampicin ± NAC; E; NO HEAT

Conditions: Matched no-heat comparison; pH not reported

Endpoint and controls: Viable-burden outcome; NAC-alone coverage partial

Do not attribute the benefit of induction heating in other arms to NAC; comparator is already multidrug.

Source: Pijls, B. G., et al. (2020); Pijls.pdf; PDF pages 3–5

## C15 | P. aeruginosa; ciprofloxacin with NAC-containing vs control coating; E

Conditions: Artificial sputum; flow 1 µL/min; mucin conditions differ

Endpoint and controls: Flow-biofilm response; coating-specific controls

A delivery/retention contrast under flow is not evidence for synergy of free NAC and ciprofloxacin.

Source: Birk, S. E., et al. (2021); Birk.pdf; PDF pages 22–24

## C16 | Catheter-derived Gram-negative isolates; six antibiotics ± NAC; E

Conditions: 24-h plate biofilm; CAMHB treatment; NAC 75 mg/mL; pH not reported

Endpoint and controls: Crystal-violet biomass; NAC-alone biofilm arm not identified

Lower author-defined MBIC indicates a biomass-associated response, not proven killing or synergy; isolates came from catheters but assay substrate was a plate.

Source: Aksoy et al. (2022); Aksoy.pdf; PDF pages 2–4

## C17 | P. aeruginosa; antibiotic selection ± NAC; R

Conditions: Flow-cell and agitated bead models

Endpoint and controls: Resistance emergence / population dynamics

NAC effects were model-dependent; do not recode as immediate antibiofilm killing.

Source: Higazy, D., et al. (2024); Higazy The impact.pdf; PDF pages 8–10

## C18 | P. aeruginosa; antibiotic selection ± NAC; R

Conditions: Synthetic CF medium 2; static aggregates; NAC 2 mM; serial passages

Endpoint and controls: Resistance trajectories rather than eradication

Conditional evolutionary effects do not establish prevention of clinical resistance.

Source: Higazy, D., et al. (2026); Higazy.pdf; PDF pages 3–5, 10–11

## C19 | Implant staphylococci; several antibiotics ± NAC; E

Conditions: NAC 1.024 mg/mL; Calgary model; historical antibiotic data for several contrasts

Endpoint and controls: MBEC; incomplete NAC-alone documentation

Historically derived comparisons are contextual, not equivalent to concurrently controlled potentiation.

Source: Molina-Manso, D., et al. (2016); Molina-Manso.pdf; PDF pages 1–2

## C20 | Rapidly growing mycobacteria; antibiotics ± NAC; E

Conditions: Middlebrook 7H9 microscopy; 80 rpm; historical components; NAC 512 mg/L

Endpoint and controls: CLSM and MBEC; arms differ by assay

Keep historical MBEC/ciprofloxacin comparisons separate from concurrent microscopy; no formation-prevention classification merely because development is measured.

Source: Munoz-Egea, M. C., et al. (2016); Munoz-Egea.pdf; PDF pages 2–4

## C21 | PAO1; ciprofloxacin-loaded cement with/without NAC; F

Conditions: 6 mg NAC and 2 g ciprofloxacin per 40 g cement; TSB + 0.5% glucose; 48 h; exposure pH/agitation not reported

Endpoint and controls: Blank, NAC-only, ciprofloxacin-only and combination cement; recovered CFU and SEM

Table I mean entries: ciprofloxacin 4 versus combination 0.01 on the reported common scale. Loading precedes inoculation; no separate established-treatment sequence. Unit/SD/SEM and SEM-score reporting inconsistencies preclude standardized effect extraction; no explicit pairwise Results statistic or formal interaction test.

Source: Onger, M. E., et al. (2016); Onger(2).pdf; PDF pages 2-4; Table I/II

## C22 | EC_219 and KP_059; dose-matched TL versus TNL; E

Conditions: Same reported tobramycin dose: EC_219 32 mg/L; KP_059 16 mg/L; 24-h treatment; preparation PBS pH 7.4, final exposure pH unreported

Endpoint and controls: Crystal-violet biomass; free NAC 50 mM control, no NAC-only liposome; lipid composition shared but size/encapsulation differ

Figure 5 EC_219 shows higher TNL than TL mean biomass; Figure 7 KP_059 shows lower TNL. TL/TNL pairwise significance is not supplied in these annotations. Do not infer uniform improvement or a validated biofilm interaction from the two directions.

Source: Alarfaj, R. E., et al. (2022); Alarafaj 2022(1).pdf; PDF pages 3-7, 10-11; Figures 5, 7

## C23 | EC_162, KP_050, RAB_005 and RAB_009; unmatched MIC-linked TL/TNL doses; E

Conditions: TL/TNL tobramycin (mg/L): EC_162 32/16; KP_050 8/16; RAB_005 128/16; RAB_009 128/16

Endpoint and controls: Crystal-violet biomass; NAC-alone free-drug control; respective formulation MICs from planktonic assays

Contextual formulation/exposure findings only: unequal antibiotic doses cannot isolate an incremental NAC effect at a common dose. Reduction versus untreated control and lower planktonic MIC do not prove biofilm killing or synergy.

Source: Alarfaj, R. E., et al. (2022); Alarafaj 2022(1).pdf; PDF pages 7, 9-12; Table 4; Figures 4, 6, 8, 9

## C24 | Seven catheter-associated isolate groups; levofloxacin lock with/without NAC; E; 12 h

Conditions: 24-h TSB colonization; saline lock NAC 10 mg/mL + levofloxacin 5 mg/mL; exposure pH not reported; no perfusion

Endpoint and controls: Concurrent saline, NAC-only, levofloxacin-only and combination; CFU/cm with SEM; nested catheter segments

Figure 1: added CFU reduction versus levofloxacin for MRSE, both MRSA phenotypes, P. aeruginosa and E. coli; VRE and K. pneumoniae comparisons marked NS. NS is not equivalence. No formal synergy model; saline control benefit is not the same as antibiotic-relative benefit.

Source: Mansouri, M. D., et al. (2022); Mansouri 2022(1).pdf; PDF pages 2-4; Figure 1; 6-7 SEM

## C25 | NAC-levofloxacin catheter-lock durability; E; 24-96 h

Conditions: Same NAC 10 mg/mL + levofloxacin 5 mg/mL in saline; extended dwell; no pH measurement or matched monotherapy arms

Endpoint and controls: Combination versus saline only; recovered CFU; no post-withdrawal regrowth test or reported quantitative detection limit

No colonies recovered for P. aeruginosa/E. coli at 24 h and MRSA at 96 h; MRSE mean approximately 5.3 x 10^3 versus 1.5 x 10^6 with saline at 96 h. These are assay-specific combination-versus-saline observations, not sustained potentiation over levofloxacin alone or proof of clinical salvage.

Source: Mansouri, M. D., et al. (2022); Mansouri 2022(1).pdf; PDF pages 3-5; Figure 2; 8

# S6. Data and analysis files

The supplementary dataset includes Study_extraction.csv, Study_phase_map.csv, Key_comparisons.csv, Full_text_exclusions.csv, Reports_not_retrieved.csv, Source_inventory.csv, selected source-reported numerical values and Analysis_summary.json. NAC_Revised_Extraction.xlsx provides the corresponding workbook, with a data dictionary and provenance records. Analysis/reproduce_analysis.py generates the descriptive counts and Figures 1–4. The 25 focused comparisons are not an exhaustive strain-by-dose arm-level dataset; missing numerical effects and variances were not imputed.
