# Supplementary material for: N-acetylcysteine as an adjunct to antibiotics against bacterial biofilms: A systematic review of in vitro evidence
Source: Biofilm. 2026 Sep 23;12:100402. doi: 10.1016/j.bioflm.2026.100402 (PMC13634462; doi:10.1016/j.bioflm.2026.100402)
Supplement: Supplementary file 2 [file mmc2.docx]

# PRISMA 2020 reporting checklist

| **Item** | **Reporting item** | **Location / status** |
| --- | --- | --- |
| 1 | Identify the report as a systematic review. | Title |
| 2 | Provide a structured summary of background, methods, results, limitations and conclusions. | Source/search limitations included |
| 3 | Describe the rationale in the context of existing knowledge. | Introduction |
| 4 | State the review objective or question explicitly. | Introduction, final paragraph |
| 5 | Specify inclusion and exclusion criteria and grouping for synthesis. | 2.2; 2.5; 2.7; Supplementary S1 |
| 6 | List databases and other sources and the date last searched. | 2.3; S2. Exact database-specific dates/logs unavailable; not fully reproducible. |
| 7 | Present full search strategies, filters and limits. | S2 presents recorded strategies and yields; execution dates and raw exports could not be verified. |
| 8 | Describe how records and reports were screened and selected. | 2.4. Independent duplicate screening not documented; automation not presented as independent review. |
| 9 | Describe how data were extracted and verified. | 2.4–2.5; S4; source inventory. No independent duplicate extraction claim. |
| 10a | Define outcomes sought and decisions about compatible results. | 2.2; 2.5; 2.7; intent/endpoint/source fields. Complete arm-level extraction not undertaken. |
| 10b | Define study, organism, intervention and model variables collected. | 2.5; S1; S4; data dictionary. Nonreporting not equated with absence. |
| 11 | Describe the appraisal method. | 2.6. Domain-based interpretability appraisal, not a validated risk-of-bias instrument or summed score. |
| 12 | Specify effect measures or direction categories. | 2.7. Original endpoint types retained; no pooled standardized measure. |
| 13a | Describe how studies were assigned to syntheses. | 2.2 and 2.7; phase map; comparator hierarchy |
| 13b | Describe transformations or handling of results. | 2.5–2.7; no imputation or figure digitization; Analysis script |
| 13c | Describe methods used to tabulate and visualize results. | 2.7; Tables 1–3; Figures 1–4; S4–S5 |
| 13d | Describe synthesis methods and rationale. | 2.7; structured narrative without efficacy vote counting or meta-analysis |
| 13e | Describe methods used to explore heterogeneity. | 2.7; 3.4–3.9; pH, medium, flow, phase, controls and regimen comparisons; descriptive, no subgroup test |
| 13f | Describe sensitivity analyses. | 2.7 and 3.10. Qualitative exclusion from strong claims of unequal-dose/historical comparisons; no pooled sensitivity estimate. |
| 14 | Describe assessment of missing results. | 4.6; no formal assessment or test |
| 15 | Describe certainty assessment. | 2.6; 4.6. No formal GRADE or certainty rating; limitations described by domain. |
| 16a | Report numbers from identification through inclusion. | 3.1; Figure 1; identification and screening totals unverified against raw exports |
| 16b | Cite or list apparently eligible reports that were excluded and explain why. | S3; Full_text_exclusions.csv |
| 17 | Present characteristics of included studies. | 3.2–3.3; Table 1; S4; extraction workbook |
| 18 | Present appraisal results. | 3.3; S4. Source/control/chemistry domains described, not validated risk-of-bias judgments. |
| 19 | Present relevant results for each study. | S4–S5; focused comparisons and outcome descriptions; not exhaustive numerical effect/precision extraction. |
| 20a | Summarize contributing studies for each synthesis. | 3.4–3.9; Tables 2–3; S4–S5 |
| 20b | Present pooled estimates if performed. | 3.1–3.10; no pooled effects |
| 20c | Present subgroup or heterogeneity findings. | 3.4–3.9; no causal subgroup effect inferred |
| 20d | Present sensitivity analyses. | 3.10; qualitative only |
| 21 | Present assessments of reporting bias. | Not statistically assessed; 4.6 |
| 22 | Present certainty assessments. | No formal certainty grading; 4.6 |
| 23a | Provide a general interpretation in context. | 4.1–4.5; Conclusions |
| 23b | Discuss limitations of included evidence. | 4.2–4.6 |
| 23c | Discuss limitations of the review process. | 2.3–2.4; 4.6; search reproducibility, source reporting, duplication and exploratory-analysis limitations |
| 23d | Discuss implications for practice, policy and research. | 4.4–4.7; Conclusions |
| 24a | Provide registration information. | 2.1; Declarations: not registered |
| 24b | Indicate where protocol can be accessed. | 2.1: no prospectively registered protocol reported; extraction framework in Supplementary S1 |
| 24c | Describe protocol amendments. | Exploratory status: 2.1 and 4.6. Methodological amendment record: Response, Appendix A. |
| 25 | Describe financial and non-financial support. | Declarations; title page |
| 26 | Declare competing interests. | Declarations; title page |
| 27 | Report availability of extraction forms, data and code. | Declarations; S6; Data/ and Analysis/ folders; original search exports and source PDFs not redistributed |
